# PGC-1α deficiency reveals sex-specific links between cardiac energy metabolism and EC-coupling during development of heart failure in mice

**DOI:** 10.1093/cvr/cvab188

**Published:** 2021-06-04

**Authors:** Nikolay Naumenko, Maija Mutikainen, Lari Holappa, Jorge L Ruas, Tomi Tuomainen, Pasi Tavi

**Affiliations:** A.I. Virtanen Institute for Molecular Sciences, University of Eastern Finland, PO Box 1627, FI-70211 Kuopio, Finland; A.I. Virtanen Institute for Molecular Sciences, University of Eastern Finland, PO Box 1627, FI-70211 Kuopio, Finland; A.I. Virtanen Institute for Molecular Sciences, University of Eastern Finland, PO Box 1627, FI-70211 Kuopio, Finland; Department of Physiology and Pharmacology Karolinska Institutet, Biomedicum, Solnavägen 9 171 65 Solna, Sweden; A.I. Virtanen Institute for Molecular Sciences, University of Eastern Finland, PO Box 1627, FI-70211 Kuopio, Finland; A.I. Virtanen Institute for Molecular Sciences, University of Eastern Finland, PO Box 1627, FI-70211 Kuopio, Finland

**Keywords:** Sex, Energy metabolism, Heart failure, t-Tubule, Local Ca^2+^ signalling

## Abstract

**Aims:**

Biological sex has fundamental effects on mammalian heart physiology and pathogenesis. While it has been established that female sex is a protective factor against most cardiovascular diseases (CVDs), this beneficial effect may involve pathways associated with cardiac energy metabolism. Our aim was to elucidate the role of transcriptional coactivator PGC-1α in sex dimorphism of heart failure (HF) development.

**Methods and results:**

Here, we show that mice deficient in cardiac expression of the peroxisome proliferator-activated receptor gamma (PPAR-γ) coactivator-1α (PGC-1α) develop dilated HF associated with changes in aerobic and anaerobic metabolism, calcium handling, cell structure, electrophysiology, as well as gene expression. These cardiac changes occur in both sexes, but female mice develop an earlier and more severe structural and functional phenotype associated with dyssynchronous local calcium release resulting from disruption of t-tubular structures of the cardiomyocytes.

**Conclusions:**

These data reveal that the integrity of the subcellular Ca^2+^ release and uptake machinery is dependent on energy metabolism and that female hearts are more prone to suffer from contractile dysfunction in conditions with compromised production of cellular energy. Furthermore, these findings suggest that PGC-1α is a central mediator of sex-specific differences in heart function and CVD susceptibility.

## Graphical Abstract

**Figure:**
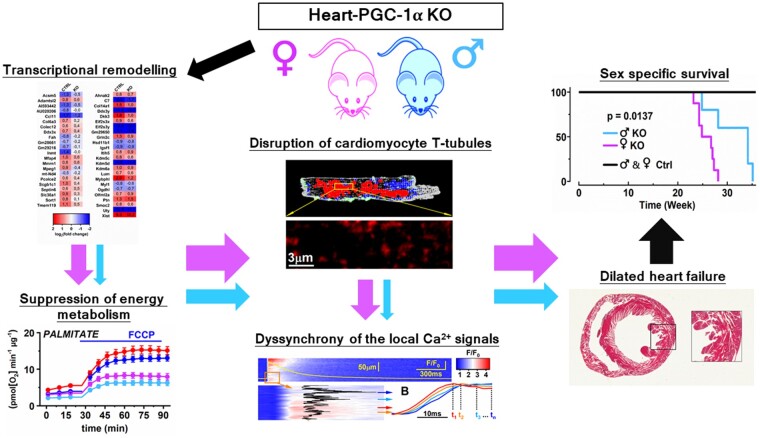

## 1. Introduction

Biological sex has a significant impact on mammalian heart physiology throughout the lifespan. In humans, sex bias has been recognized in the prevalence and outcome of various cardiac diseases^1^ and sex dimorphism has been recapitulated in animal disease models.^2^^,^^3^ Sex dimorphism is manifested as a spectrum of physiological specifications of the heart, including size,^4^ contractile performance,^5^ and energy metabolism^6–8^ and at the level of individual cardiomyocytes calcium signalling^5^ and electrophysiology.^9^

While the overall biological lifetime risk for cardiovascular diseases (CVDs) is similar between sexes, men develop diseases earlier than women.^10^ The combined effect of female chromosomes and oestrogen reduces the risk of atherosclerosis, ischaemic heart disease, and hypertension.^11^ While female sex is a protecting factor against most cardiac diseases of pre-menopausal females,^4^ some reports suggest that female sex is associated specifically with greater prevalence or worse prognosis in metabolic cardiomyopathies^8^ and hypertrophic cardiomyopathy.^12^^,^^13^

A central difference in cardiac pathophysiology between sexes is that compared to males, females develop more favourable forms of hypertrophy associated with beneficial adaptation of cardiac energy metabolism^14^ in response to either physiological or pathological cardiac load.^15^ It can be hypothesized that pathways mediating metabolic plasticity in females might be central in explaining the sex disparities in CVDs. Metabolic plasticity of the heart involves central metabolic pathways consisting of transcriptional coactivator peroxisome proliferator-activated receptor gamma (PPAR-γ) coactivator-1α (PGC-1α) and its transcription factor partners and downstream targets PPARα,δ, oestrogen-related receptors (ERRα,γ), and nuclear respiratory factors 1, 2.^16^^,^^17^ A line of evidence suggests that PGC-1α/PPAR and their target genes might be involved in sex disparity of cardiac energy metabolism and pathogenesis.^8^ Sex-specific cardiac PPARα expression^18^ and sex-specific effects of PPARα deletion in the heart have been reported.^19^ On the other hand, oestrogen has been shown to regulate PGC-1α expression in liver^20^ and heart,^21^^,^^22^ which has partly initiated the idea that PGC-1α might be central in protecting female hearts against pathological developments.^23^ This sexual dimorphism might be relevant in cardiac pathogenesis since female mice have been found to be more resistant to pressure load-induced PGC-1α downregulation^24^ and PPARα-centred gene expression pattern was identified to mediate the sex differences in the development of hypertrophy in mice.^25^ In humans, pressure overload triggers adaptive upregulation of genes involved in peroxisome-dependent utilization of lipids in female left ventricles, whereas in male hearts genes involved in oxidative phosphorylation are downregulated.^26^

We hypothesized that the progression of diseases associated with compromised myocardial energy metabolism might reveal some of the mechanisms behind sexual dimorphism in CVDs. To test this hypothesis, we studied mice deficient in cardiac PGC-1α, a model that recapitulates the energy metabolic changes associated with the development of heart failure (HF) including gradual suppression of energy metabolism and parallel decline of the contractile function leading to HF. We demonstrate that female Heart-PGC-1α KO mice develop HF earlier than male mice due to earlier development of severe contractile dysfunction and specific changes in the regulation of subcellular Ca^2+^ release and uptake resulting from disruption of transverse tubules (t-tubules). This work sets PGC-1α as a central mediator of sex-specific differences in heart function and CVD susceptibility.

## 2. Methods

All detailed method sections are provided in the Supplementary material online.

### 2.1 Ethical approval

All animal experiments were carried out with authorization by The National Animal Experiment Board of Finland (animal experimentation permit # ESAVI/7867/2018) and following the guidelines of The Finnish Act on Animal Experimentation, which comply with the guidelines from Directive 2010/63/EU of the European Parliament on the protection of animals.

### 2.2 Experimental animals

Male and female mice homozygous for floxed *Pgc-1α* and hemizygous for Myh6-Cre (Heart-PGC-1α KO)^27^ were used in experiments. Age-matched littermates homozygous for floxed *Pgc-1α* and negative for Myh6-Cre were used as a control. For the collection of tissues, animals were euthanized with CO_2_ inhalation, followed by cervical dislocation. For cell isolation, animals were euthanized by cervical dislocation without preceding CO_2_ inhalation.

### 2.3 Survival follow-up

Heart-PGC-1α KO mice were aged as long as they did not show visually observable signs of impaired cardiac function, which were considered as time points for euthanasia.

### 2.4 Echocardiography

For echocardiography, a high-resolution Vevo2100 Ultrasound imaging system (Visual Sonics Inc., Toronto, Canada) was used as described previously.^28^ Animals at the ages of 10, 12, and 16 weeks were imaged under inhalation anaesthesia [induction with 3.5–4% isoflurane (Baxter International Inc., Deerfield, IL, USA) and 350–400 mL/min air, maintenance with 2–2.5% isoflurane and 200–250 mL/min air] and kept on a heated platform during and shortly after the imaging.

### 2.5 RNA sequencing

Total RNA from left ventricular tissue of 18-week-old mice was isolated with TRI reagent (Sigma-Aldrich) using TissueLyzer II (Qiagen) in homogenization. Library preparation and sequencing were performed in the Finnish Functional Genomics Centre (Turku, Finland). RNA sequencing libraries were prepared from 300 ng total RNA according to Illumina TruSeq Stranded mRNA Sample Preparation Guide (#15031047). Libraries were sequenced with Illumina HiSeq 3000 instrument using 50 bp single-end sequencing and sequencing data were analysed with software^29–32^ described in the Supplementary material online.

### 2.6 Quantitative RT-PCR

cDNA was synthesized from ventricular total RNA of 18- and 22-week-old mice with the RevertAid First Strand cDNA Synthesis Kit (ThermoFisher Scientific, Waltham, MA, USA). TaqMan-based quantitative PCR was performed with StepOnePlus™ Real-Time PCR System (Applied Biosystems, Foster City, CA, USA). Sequences of the fluorogenic probes and primers are presented in Supplementary material online, *Table S1*. Expression level of *hypoxanthine-guanine phosphoribosyltransferase* gene was used in normalization.

### 2.7 Single-cell isolation

Adult cardiomyocytes were isolated from 18-week-old mice or from 22-week-old animals as described previously.^33^

### 2.8 Analysis of energy metabolism in isolated cardiomyocytes

Energy metabolism was assessed with Seahorse XF24 Analyzer (Agilent Technologies) as described earlier.^27^

### 2.9 Confocal imaging of isolated cardiomyocytes

For calcium and t-tubule imaging the confocal imaging system (FluoView 1000, Olympus, Japan) was used. Cytosolic calcium signals in Fluo4-loaded cells were measured as described previously.^33^ For t-tubule staining CellMask Orange was used. Images were analysed using FluoView 4.0 (Olympus, Japan), ImageJ 1.5 (https://imagej.nih.gov/ij/), and MatchedMyo software.^34^

### 2.10 Ca^2+^ flux protocol

To estimate calcium fluxes, Fluo4-loaded cardiomyocytes were exposed to Tyrode-based solutions with different compositions (*Figure 5A*). Sarcoplasmic reticulum (SR) calcium leak through ryanodine receptors (RyRs) was estimated in 0 Ca^2+^ and 0 Na^+^ solution with tetracaine.^35^ SR calcium content was assessed by caffeine application and SR Ca^2+^-ATPase activity (SERCA) by single exponential fitting of caffeine-induced Ca^2+^ transient decay. Second extended caffeine pulse was used to measure the plasma membrane Ca^2+^ ATPase activity (PMCA). Restoring to normal [Na^+^]_o_ and [Ca^2+^]_o_ in this condition led to accelerating of decay which was used to quantify sodium–calcium exchanger activity.

### 2.11 Spatiotemporal characteristics of calcium release

For detailed assessment of spatial and temporal dyssynchrony of calcium release in Fluo4-loaded cardiomyocytes, we analysed time course parameters of the local calcium transient (locCaT) at subcellular level. Briefly, fluorescence intensity was analysed for every single pixel of the original line-scans. The degree of locCaT dyssynchrony was assessed as the standard deviation (σ) in the time course of locCaTs within the cell.^36^ For spatial-σ, only one whole cell calcium transient (CaT) was included into the analysis. To measure temporal-σ (*Figure 6F*) five consecutive CaTs were analysed^36^^,^^37^ and to evaluate structural-σ, we eliminated beat-to-beat deviation in calcium release by averaging five frames of original line-scan with five consecutive CaTs as described previously.^37^^,^^38^

### 2.12 Whole cell patch-clamp

For ionic currents and action potential (AP) recordings, a patch-clamp amplifier Axopatch 200B in combination with a Digidata 1440 A and Clampex 10 software (Molecular Devices Inc., Sunnyvale, CA, USA) were used. Protocols for AP,^33^ L-type Ca^2+^-current,^39^ and potassium currents^39^ recordings were as described previously.^33^ Analysis of the recordings was performed with ClampFit 10 (Molecular Devices).

### 2.13 Histology and western blot

Histology and western blot protocols are described in the Supplementary material online.

### 2.14 Statistical testing

OriginPro program (OriginLab Corporation, Northampton, MA, USA) was used for statistical analysis. Results are presented as mean ± standard error of the mean. Hierarchical linear model was used for data from experiments with isolated cardiomyocytes.^40^ In other cases, statistical significance and interaction between sex and genotype were estimated with two-way ANOVA followed by Bonferroni *post hoc* test.

## 3. Results

### 3.1 PGC-1α deficiency-induced HF shows sexual dimorphism

**Figure 1:**
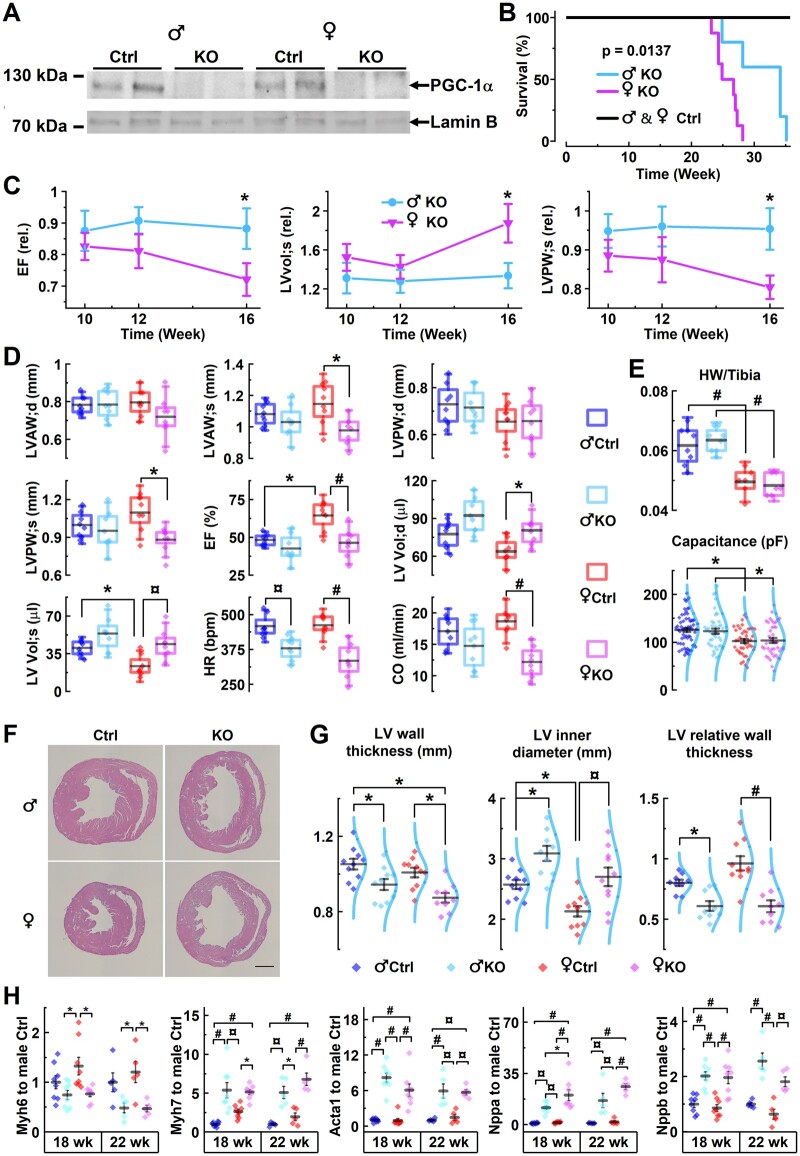
PGC-1α knockout causes sex-specific gradual cardiac dilatation and contractile suppression. (*A*) PGC-1α western blot from ventricular tissue of male and female Heart-PGC-1α KO and control mice. (*B*) Survival of male and female KO and control mice during a 250-day follow-up period (male Ctrl *n* = 5, male KO *n* = 5, female Ctrl *n* = 10, female KO *n* = 8). (*C*) Ejection fraction (EF), systolic left ventricular volume (LVvol; s), and systolic left ventricular posterior wall thickness (LVPW; s) of male and female KO mice shown as relative to average value in age-matched littermate controls at the ages of 10, 12, and 16 weeks (*n* = 8). (*D*) Parameters from echocardiography of 16-week-old male and female KO and control mice. CO, cardiac output; d, diastole; EF(%), ejection fraction shown as percentage; HR, heart rate; LVAW, left ventricular anterior wall thickness; LVPW, left ventricular posterior wall thickness; LV_vol_, left ventricular volume; s, systole (*n* = 8). (*E*) Heart weight to tibial bone length ratio (upper) (males *n* = 8, females *n* = 6) at the age of 16 weeks and cell capacitance at the age of 18 weeks [lower panel; male Ctrl *n* = 11/69 (animals/cells), male KO *n* = 10/53, female Ctrl *n* = 8/47, and female KO *n* = 8/43]. (*F*) Representative haematoxylin–eosin stained cross-sections of 21-week-old male and female mice hearts (scale bar 1 mm). (*G*) Ventricle wall thickness, inner perimeter derived diameter, and relative wall thickness of left ventricles measured from the histological sections (*n* = 9) of 21-week-old mice. (*H*) Ventricular expression of heart failure marker genes at the ages of 18 (*n* = 8) and 22 (*n* = 5) weeks (refer to Supplementary material online, *Table S1* for gene abbreviations). Statistical significance from hierarchical linear model (*E*) and ANOVA Bonferroni *post hoc* test (*B*–*D, G*, and *H*); *P < 0.05, ^¤^*P* < 0.01, ^#^*P* < 0.001.

*Pgc-1α* ^fl/fl^ + Myh6-Cre (Heart-PGC-1α KO) mice have normal PGC-1α expression in other tissues except for the heart, which completely lacks PGC-1α expression^27^ in both male and female animals (*Figure 1A*). At birth, PGC-1α KO animals were undistinguishable from littermate controls but by 16 weeks of age both sexes showed signs of developing cardiomyopathy, manifested as premature death from 23 weeks onwards in females, and 25 weeks onwards in males with a significant difference between sexes (*Figure 1B*). During the period preceding the premature death of the animals, from 10 to 16 weeks of age, cardiac function developed differently between males and females. Up to 16 weeks of age male KO mice showed very small changes, compared to their age-matched controls, in ejection fraction (EF), left ventricular volume (LV_vol_), or LV wall thickness. Female animals, on the other hand, showed significant decreases in EF and LV wall thickness as well as an increase in LV_vol_ compared to control animals (*Figure 1C and D*). At the 16 weeks of age, control mice showed physiological sex-dependent differences in the LV_vol_ and EF (*Figure 1D*). At this time point, PGC-1α deficiency affected almost exclusively female animals, which showed clear signs of dilated HF indicated by decrease of the EF, increase in left ventricular volume (LV Vol; s and LV Vol; d) and thinning of anterior and posterior walls in systole, together resulting in reduced cardiac output (CO; *Figure 1D*).

Female mice had smaller heart and cardiomyocyte sizes, and the same difference was present in PGC-1α KO animals (*Figure 1E*). It appears that PGC-1α KO mice have no hypertrophy but instead the phenotype is associated with LV dilatation, which is more pronounced in female animals (*Figure 1F and G* and Supplementary material online, *Figure* *S1*). Histological cross-sections showed slight changes in PGC-1α KO hearts already at the age of 12 weeks and LV dilatation was apparent at the age of 18 weeks especially in female KO mice (Supplementary material online, *Figure* *S1*). At the age of 21 weeks signs of LV dilatations were even more pronounced as the LV wall thickness was significantly reduced, and the LV inner diameter was increased in both male and female KO animals (*Figure 1G*). As a result, PGC-1α KO animals had significantly reduced relative wall thickness, and change was more pronounced in females (*Figure 1F and G*). These structural changes were associated with similar changes in expression of HF marker genes in both female and male PGC-1α KO hearts at the ages of 18 and 22 weeks, except for α-myosin heavy chain (Myh6) which was reduced only in female PGC-1α KO hearts (*Figure 1H*).

### 3.2 PGC-1α deficiency-induced transcriptional remodelling is similar between sexes

**Figure 2:**
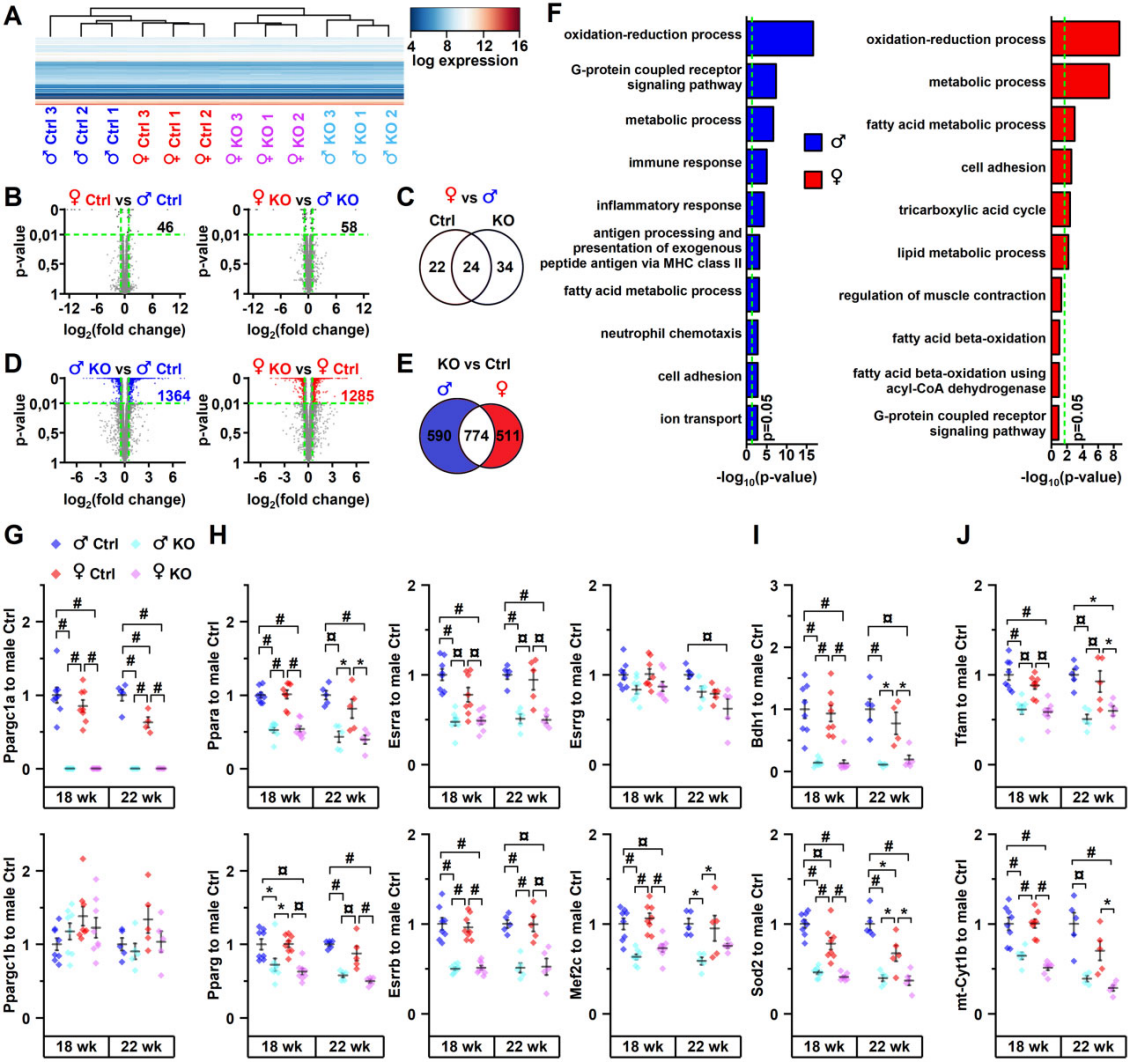
PGC-1α knockout induces similar transcriptional changes in male and female hearts. (*A*) Heatmap showing logarithmic expression level of all 11 970 genes passing the expression level cut-off as assessed with RNA sequencing from 18-week-old animals (*n* = 3). Hierarchical clustering was applied for rows (genes) and columns (samples). (*B*) Sex differences in gene expression between control (left) and heart-specific PGC-1α knockout (right) mice. Log_2_ transformed fold change of each gene is plotted against adjusted *P*-value. Dots for genes passing the differential expression cut-offs [shown by dashed lines: *P*-value 0.01, log_2_ (fold change) ± 0.5) are highlighted with blue and red. Note that *y*-axis is stretched between *P*-values 0.01 and 0. (*C*) Venn diagram showing the overlap of gender differences in gene expression between control and knockout mice. (*D*) Gene expression changes induced by heart-specific PGC-1α knockout in male (left) and female (right) mice. (*E*) Venn diagram showing the overlap of differentially expressed genes in male and female mice. (*F*) Gene enrichment analysis (GO: biological process) for the sets of differentially expressed genes between male and female highlighted in (*D*). Bonferroni corrected *P*-values for top 10 enriched terms are shown. Dashed line corresponds to *P*-value 0.05. (*G*–*J*) Ventricular gene expression of PGC-1α/β (*G*), transcription factors co-activated by PGC-1α (*H*), known PGC-1α target genes (*I*), and genes related to mitochondrial function (*J*) at the ages of 18 (*n* = 8) and 22 (*n* = 5) weeks (refer to Supplementary material online, *Table S1* for gene abbreviations). In Ppargc1a and Bdh1 22-week-old female control *n* = 4. ANOVA Bonferroni *post hoc* test: *P < 0.05, ^¤^*P* < 0.01, ^#^*P* < 0.001.

To get a better view of the overall changes induced by the PGC-1α knockout in male and female hearts, we performed RNA sequencing from left ventricular tissues. Hierarchical clustering of samples based on expression of 11 970 genes passing the expression level filter (Supplementary material online, *Figure* *S2A*) showed that the samples were segregated by the genotype and further by sex (*Figure 2A*), which implicates the genotype as the main factor affecting ventricular transcriptome. Supporting this, principal component analysis led to a similar clustering of samples (Supplementary material online, *Figure* *S2B*). When comparing the sex differences, 46 genes had different expression between control males and females (*Figure 2B*, left). In the KO hearts, 58 genes had different expression level between sexes (*Figure 2B*, right), from which 24 genes maintained the sex difference present in control animals and 34 genes acquired significant difference (*Figure 2C*). Genes with differential expression between males and females are shown in heatmap in Supplementary material online, *Figure* *S2C*. In the gene set enrichment analysis, no gene ontology (GO) biological processes were significantly enriched among the genes with sex difference (Supplementary material online, *Table S2*). Comparison between control and KO hearts revealed differential expression of 1364 genes in males and 1285 in females (*Figure 2D*) with considerable overlap between the genes affected in males and females (*Figure 2E*). As expected, in both sexes GO biological processes related to oxidative metabolism were among the top enriched ones (*Figure 2F* and Supplementary material online, *Table S2*). Lack of PGC-1α had also different effect on many transcripts between males and females (*Figure 2E*), which led to some differences in the enriched gene pathways (*Figure 2F*). Further inspection of genes passing the differential expression filter only in either of the sexes reveals similar expression pattern in the opposite sex (Supplementary material online, *Figure* *S2D* *and* *E*), which explains the exceedingly small changes in sex differences between controls and knockouts (*Figure 2C*). In addition, clustering of samples is not changed dramatically when it is performed with these subsets of genes (Supplementary material online, *Figure* *S2D* *and* *E*) compared to when all genes are used (*Figure 2A*). A similar trend is seen when genes belonging to specific pathways enriched only in either of the sexes are analysed (Supplementary material online, *Figure* *S2F* and *G*). We also measured gene expression changes from the members of PGC-1α cascade to see if the differential survival between sexes could be explained by their differential regulation. We quantified expression changes at the age of 18 weeks and at the age of 22 weeks, which is closer to the time point where survival of males and females starts to differ (*Figure 1B*). PGC-1α knockout was not compensated in either of the sexes and in either of the time points by PGC-1β gene (*Ppargc1b*) expression (*Figure 2G*). Furthermore, gene expression of transcription factors (PPARs, ORRs, MEF2) known to be co-activated by PGC-1α in cardiac myocytes were reduced to similar extent in males and females in both time points (*Figure 2H*). Expression of known PGC-1α target genes *3-hydroxybutyrate dehydrogenase 1*^41^ (*Bdh1*) and *superoxide dismutase 2*^42^ (*Sod2*) were robustly decreased in both sexes and time points (*Figure 2I*). Finally, *mitochondrial transcription factor A* (*Tfam*) and *cytochrome b* (*mt-Cytb*) were both similarly reduced in all the groups suggesting similar repression of mitochondrial function in males and females (*Figure 2J*). Analysis of gene expression indicates that males and females respond similarly to PGC-1α deficiency both in the level of overall transcriptome and in the level of individual genes belonging to the PGC-1α cascade.

### 3.3 PGC-1α deficiency suppresses cardiomyocyte energy metabolism in both sexes

**Figure 3:**
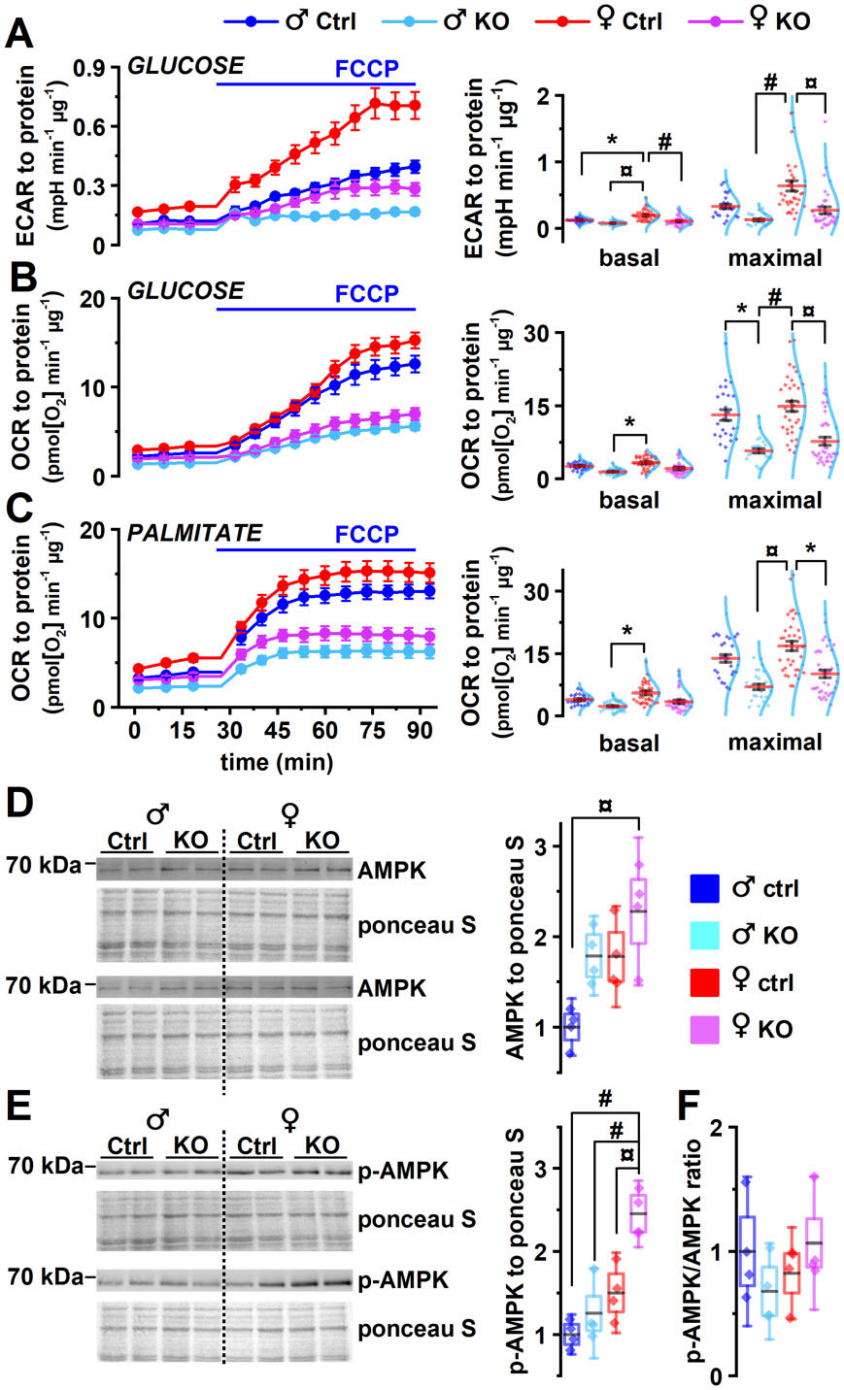
PGC-1α deficiency suppresses cardiomyocyte energy metabolism. (*A*–*C*) Cells isolated from 18-week-old animals were supplemented with different energy substrates, and basal and FCCP induced maximal energy consumption were determined. (*A*) Averaged extracellular acidification rate (ECAR) traces (left) and analysis of basal and maximal ECAR (right) in cells supplemented with glucose. (*B*) Averaged oxygen consumption rate (OCR) traces (left) and analysis of basal and maximal OCR (right) in cells supplemented with glucose. (*C*) Averaged OCR traces (left) and analysis of basal and maximal OCR (right) in cells supplemented with palmitate. (*D*) Western blot analysis (left) and quantification (right) of 5′ AMP-activated protein kinase (AMPK) in left ventricular tissue from 18-week-old animals. (*E*) Western blot analysis (left) and quantification (right) of Thr172 phosphorylated AMPK in left ventricular tissue. (*F*) Ratio of phosphorylated AMPK and total AMPK in left ventricular tissue. In (*A*–*C*), male Ctrl *n* = 5/25 (animals/seahorse wells), male KO *n* = 5/25, female Ctrl *n* = 7/35, female KO *n* = 7/35. In (*D*–*F*), *n* = 4. Statistical significance from hierarchical linear model (*A*–*C*) and ANOVA Bonferroni *post hoc* test (*D* and *E*): *P < 0.05, ^¤^*P* < 0.01, ^#^*P* < 0.001.

To investigate the energy metabolic capacity and substrate specificity of control and KO cardiomyocytes, we conducted *in vitro* metabolic analysis using Seahorse XF cell analyser. Overall, glycolytic capacity (extracellular acidification rate) in the presence of glucose (*Figure 3A*) and aerobic capacity (oxygen consumption rate) both in the presence of glucose (*Figure 3B*) or palmitate (*Figure 3C*) were suppressed in the KO cardiomyocytes of both sexes. Lack of PGC-1α had more pronounced effects on female cardiomyocyte glycolytic capacity compared to male cells (*Figure 3A*). No significant interaction between sex and genotype was detected in energy substrate preference. Activation of 5′ AMP-activated protein kinase (AMPK) was assessed to see whether KO hearts suffer from energy deprivation. Total AMPK was increased in female KO when compared to male control (*Figure 3D*). Similarly, the amount of AMPK phosphorylated at residue Thr172, indicating AMPK activation, was increased in female KO heart in comparison to all other experimental groups suggesting that PGC-1α deficiency has more severe effects on cardiac energy production in females (*Figure 3E*). The ratio of phosphorylated and total AMPK did not differ among the experimental groups (*Figure 3F*).

### 3.4 PGC-1α deletion diminishes the sex disparity of electrophysiology parameters

**Figure 4:**
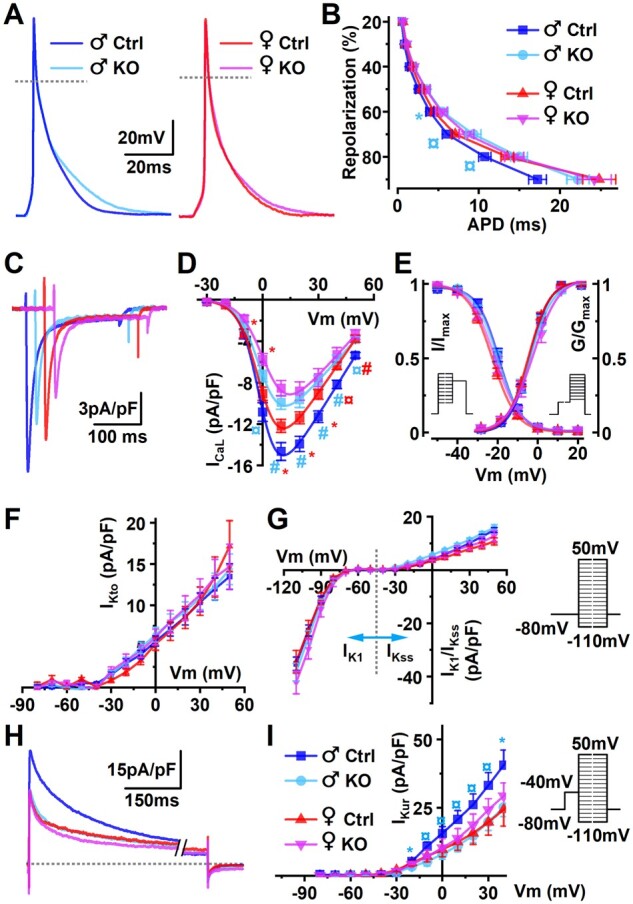
Effect of PGC-1α knockout on electrophysiological properties of isolated ventricular cardiomyocytes. (*A*) Representative superimposed action potential traces of male and female cardiomyocytes (left and right, respectively) isolated at the age of 18 weeks. (*B*) Action potential durations at different points of repolarization [male Ctrl *n* = 8/25 (animals/cells), male KO *n* = 7/22, female Ctrl *n* = 5/22, and female KO *n* = 5/20]. (*C*) Representative L-type calcium current (I_CaL_) traces obtained at +10 mV voltage step. (*D*) Voltage–current relationship of I_CaL_. (*E*) Steady-state activation (G/G_max_) and inactivation (I/I_max_) curves of I_CaL_ (male Ctrl *n* = 3/16, male KO *n* = 3/11, female Ctrl *n* = 3/16, and female KO *n* = 3/8). Inset shows voltage step protocols for activation and inactivation voltage dependencies. (*F*) Transient outward potassium currents (I_Kto_). (*G*) I_K1_ and steady-state (I_Kss_) potassium currents. (*H*) Representative traces of ultra-rapid potassium current (I_Kur_). (*I*) Voltage–current relationship of I_Kur_ (male Ctrl *n* = 8/30, male KO *n* = 7/32, female Ctrl *n* = 5/10, and female KO *n* = 4/17). Inset shows voltage step protocols for isolating potassium currents I_Kto_, I_K1_, I_Kss_, and I_Kur_ from hierarchical linear model significances shown from male Ctrl; *P < 0.05, ^¤^*P* < 0.01, ^#^*P* < 0.001.

Sex-specific features in cardiac electrophysiology give rise to sex-specific physiological features ^43^^,^^44^ and contribute to sex disparity of the disease progression.^3^ Accordingly, in control animal myocytes females had longer APs (*Figure 4A and B*) than males as described before.^44^^,^^45^ Despite that expression of Na^+^/K^+^-ATPase (*Atp1a1*) and voltage activated Na^+^-channel subunit (*Scn5a*) were reduced by PGC-1α deficiency in both sexes (Supplementary material online, *Figure* *S3A*), PGC-1α KO did not affect resting membrane potential or AP amplitudes (Supplementary material online, *Table S3*). PGC-1α deficiency did increase the AP duration at 60–80% of repolarization in males, whereas no difference between female KO and female control was detected (*Figure 4A and B*).

Male cardiomyocytes had higher calcium current densities (I_CaL_; *Figure 4C and D*) compared to females, but this sex difference was abolished in KO myocytes without alteration in voltage-dependent activation/inactivation (*Figure 4E*). These changes were accompanied by downregulation of *Cacna1c* in both male and female KO animals (Supplementary material online, *Figure* *S3A*).

Among the measured potassium currents, control males had higher density of ultra-rapid delayer rectifier K^+^ current (I_Kur_) which was reduced in KO males, equalizing the sex differences in I_Kur_ between KO males and KO females (*Figure 4H and I*). Transient outward current (I_Kto_; *Figure 4F*), inward-rectifier (I_K1_; *Figure 4G*), and steady-state potassium current (I_Kss_; *Figure 4G*) were not different between sexes nor were they changed upon PGC-1α deletion. Expression of genes encoding potassium channel subunits was affected by PGC-1α deficiency, but the response showed no sex disparity, and transcripts were similarly changed in both male and female KO hearts ranging from suppressed expression (*Kcna5, Kcnd2, Kcnj2*, and *Kcnb1*), to unaltered (*Kcna4*) and upregulated (*Kcne1*) (Supplementary material online, *Figure* *S3A*). Overall, PGC-1α deficiency equalized the sex-dependent electrophysiological differences between males and females at the level of measured potassium currents, calcium currents, and AP length.

### 3.5 PGC-1α deficiency has sexually dimorphic effects on cardiomyocyte Ca^2+^ signalling

**Figure 5:**
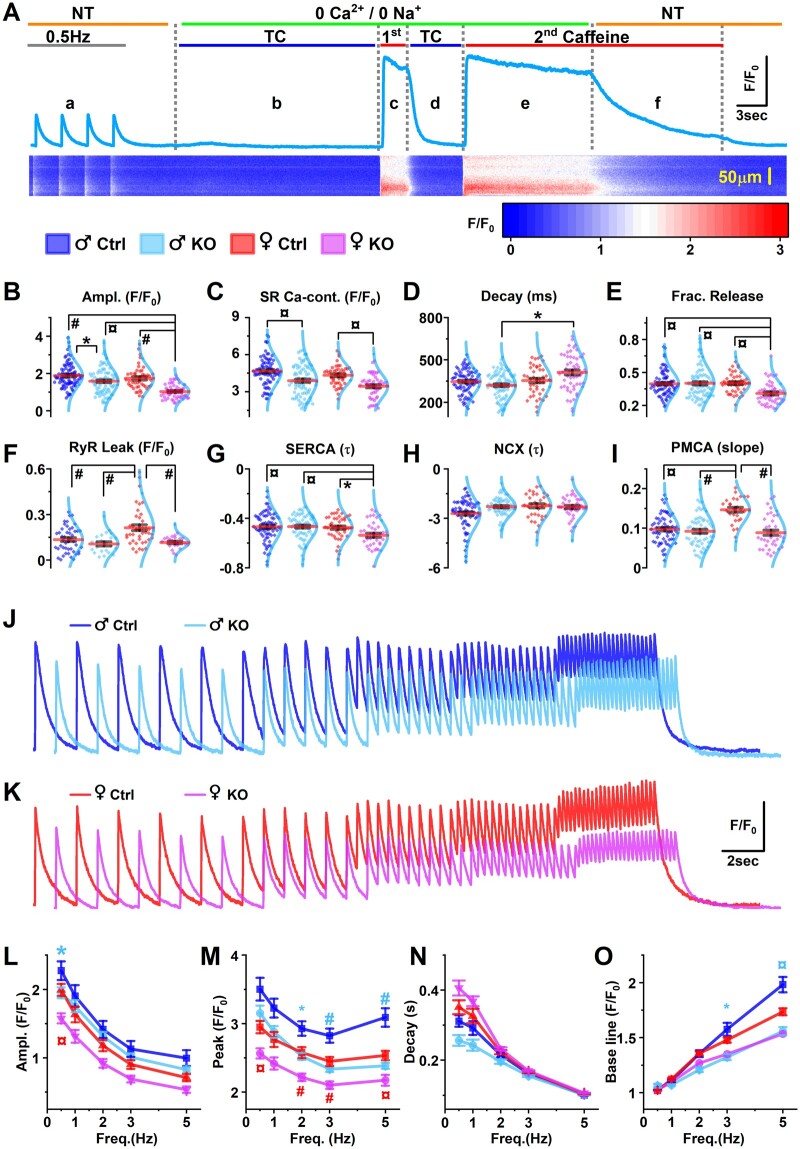
PGC-1α deficiency disrupts Ca^2+^-handling in female cardiomyocytes. (*A*) Graphical representation of sarcoplasmic reticulum (SR) Ca^2+^ fluxes estimation protocol [see details in Section 2; brief epochs description: epoch a—basic Ca^2+^ transient (CaT) parameters, epoch b—ryanodine receptor (RyR) Ca^2+^ leak, epoch c—SR Ca^2+^ content, epoch d—SR calcium ATPase (SERCA) activity, epoch e—plasma membrane calcium ATPase (PMCA) activity, epoch f—Na^+^/Ca^2+^ exchanger (NCX) activity; NT, normal Tyrode solution; TC, tetracaine). (*B*–*E*) CaT properties of cardiomyocytes isolated from 18-week-old mice [male Ctrl *n* = 4/89 (animals/cells), male KO *n* = 4/88, female Ctrl *n* = 3/47. and female KO *n* = 3/56]: (*B*) CaT amplitude, (*C*) SR Ca^2+^ content, (*D*) CaT decay, (*E*) fractional Ca^2+^ release. (*F*–*I*) Estimated Ca^2+^ fluxes: (*F*) RyR Ca^2+^ leak, (*G*) SERCA activity, (*H*) NCX activity, (*I*) PMCA activity. In (*G* and *H*), τ from single exponential fit. In (*I*), slope from linear fit. In scatter charts red line depicts mean, whiskers SEM and cyan line distribution. Representative calcium transients from male (*J*) and female (*K*) cardiomyocytes in response to 0.5, 1, 2, 3, and 5 Hz electrical stimulation. (*L*) Means of the CaT amplitudes, (*M*) peaks, (*N*) decay time 66% (decay), and (*O*) baseline of CaT evoked by frequent electrical stimulation (male Ctrl *n* = 5/24, male KO *n* = 4/31, female Ctrl *n* = 3/28, and female KO *n* = 3/29). Hierarchical linear model significance shown pairwise Ctrl vs. KO; *P < 0.05, ^¤^*P* < 0.01 and ^#^*P* < 0.001.

To estimate whether the functional and structural changes seen in female PGC-1α KO hearts are associated with disturbances in cellular calcium cycling, we employed a specific protocol designed to isolate different calcium extrusion and uptake mechanisms from each other (*Figure 5A*). This analysis revealed some sex disparity in cardiomyocyte Ca^2+^ handling, namely, female control myocytes showed higher PMCA activity and RyR Ca^2+^-leak compared to male control myocytes. Cardiomyocytes isolated from PGC-1α deficient hearts of both sexes had lower CaT amplitudes and reduced sarcoplasmic (SR) Ca-stores compared to their controls (*Figure 5B and C*). The changes were accompanied by lower expression of RyR (*Ryr2*) in both male and female KO animals and reduced calsequestrin 2 (*Casq2*) in female KO (Supplementary material online, *Figure* *S3B*). When calcium signals of male and female KO myocytes were compared, female myocytes showed smaller CaT amplitudes and longer CaT decays (*Figure 5B and D*), the latter of which indicates insufficient Ca^2+^ removal from the cytosol. Accordingly, in female PGC-1α-KO cardiomyocytes SERCA activity was the lowest among all four groups tested (*Figure 5G*). This change was not directly correlated with expression of SR Ca-ATPase SERCA (*Atp2a2*) and its inhibitory protein phospholamban (*Pln*). Although *Atp2a2* was downregulated in both sexes by PGC-1α deficiency, *Atp2a2* to *Pln* ratio did not differ between groups (Supplementary material online, *Figure* *S3B*). These changes were associated with significantly lower fractional Ca^2+^ release (SR Ca content/CaT amplitude) in female KO myocytes (*Figure 5E*), even with equal calcium current density compared to male KO cardiomyocytes (*Figure 4D*).

PGC-1α-deficient cardiomyocytes of both sexes adapt to increased frequency of pacing by increased speed of CaT decay (*Figure 5N*). However, despite the similar frequency-dependent acceleration of the CaT decay, at higher pacing frequencies female KO cardiomyocytes retained lower peaks of CaT compared to male KO at similar diastolic [Ca^2+^]_i_ (*Figure 5M*). Collectively these data indicate that PGC-1α deficiency induces sex dimorphic pathological changes in calcium signalling that are more profound in female cardiomyocytes.

### 3.6 PGC-1α deficiency disrupts the local Ca^2+^ release and uptake synchronization in female cardiomyocytes

**Figure 6:**
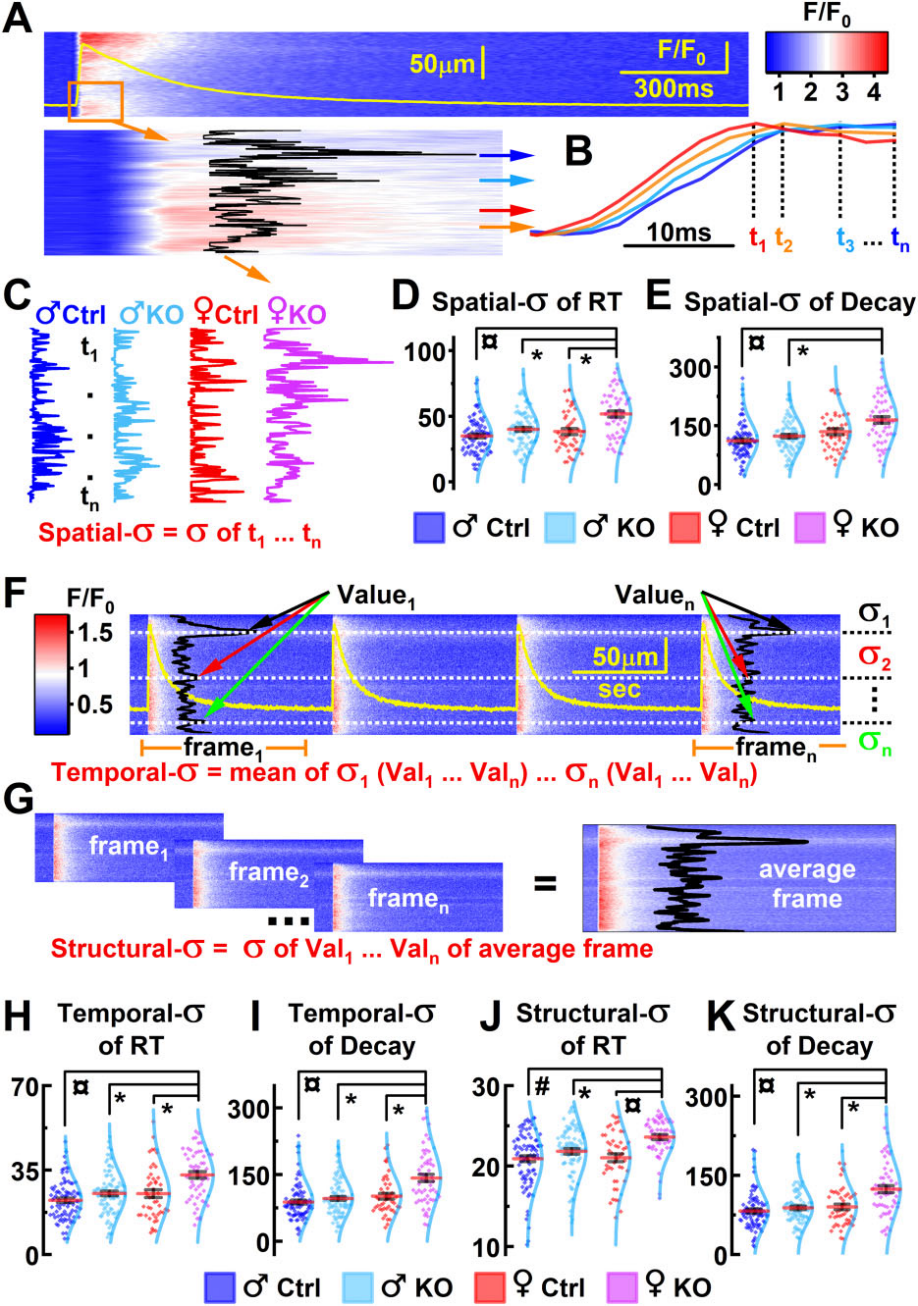
PGC-1α deficiency induces spatiotemporal dyssynchrony of local calcium release in female myocytes. (*A*) Graphical explanation of workflow for spatial dyssynchrony (spatial-σ) estimation. Yellow line is corresponding to global CaT profile in the original line-scan recording. In the zoomed in analysis region, black curve represents a profile of time-to-peak obtained from individual pixels of the line-scan. (*B*) Some representative traces of local CaTs [obtained at pixels shown by coloured arrows in (*A*); t_1_, t_2_, …, t_*n*_ is time-to-peak]. (*C*) Representative profiles of time-to-peak from which standard deviations were calculated. (*D*) Spatial-σ of CaT rise time. (*E*) Spatial-σ of CaT decay time. (*F*) Graphical explanation of beat-to-beat dyssynchrony (temporal-σ) estimation. Yellow line is corresponding to global CaT profile in the original line-scan recording. Black curves represent profiles of local CaT decays and arrows show measured values (Val_1_, …, Val_*n*_) from which corresponding pixels’ standard deviations (σ_1_, …, σ_*n*_) were calculated. Mean of each pixel’s standard deviations was considered as temporal-σ of the cell. (*G*) Graphical explanation of structural dyssynchrony (structural-σ) estimation. Averaged line-scan frame was acquired from consecutive CaT frames and analysed as in spatial-σ estimation. (*H*) Temporal-σ of CaT rise time. (*I*) Temporal-σ of CaT decay time. (*J*) Structural-σ of CaT rise time. (*K*) Structural-σ of CaT decay time. In scatter charts red line depicts mean, whiskers SEM and cyan line distribution. Eighteen-week-old animals; Ctrl *n* = 4/86 (animals/cells), male KO *n* = 4/88, female Ctrl *n* = 3/47, and female KO *n* = 3/56. Hierarchical linear model significance: *P < 0.05 and ^¤^*P* < 0.001.

Growing body of evidence suggests that pathological cardiac remodelling involves changes in cardiomyocyte substructures, t-tubules, calcium release units (CRUs), and coupling of L-type calcium channels (LTCCs) with RyRs.^46^ According to our data, female PGC-1α-KO cardiomyocytes show changes in their whole cell calcium signals, such as prolongation of rise time (RT) of CaT (Supplementary material online, *Figure* *S4*), slow CaT decay, reduced CaT amplitude (*Figure 5B*), and impaired calcium release at high pacing frequencies (*Figure 5M*). These changes might partly originate from dyssynchronized calcium release and uptake at subcellular level leading to variability in calcium signals and contributing to HF.^47–49^ A certain degree of variation in cardiomyocyte Ca^2+^ signals is part of normal physiology. For example, the variation in the locCaT amplitude closely follows the SR Ca^2+^ content (*Figure 5B* and Supplementary material online, *Figure* *S5A*) resulting in higher variability with higher SR content and Ca^2+^ release gain^50^ in male cardiomyocytes (Supplementary material online, *Figure* *S5A*). To evaluate dyssynchrony at subcellular level, we employed a pixel (0.397 µm) level analysis on the line-scanned calcium recordings (*Figure 6*). Spatial dyssynchrony measured as spatial variance (spatial-σ) of the RTs of the local calcium signals (*Figure 6A–D*), indicating the degree of dyssynchrony in the local Ca^2+^ releases, was found to be significantly higher in female KO cardiomyocytes compared to all other groups (*Figure 6D*). Similarly, female KO myocytes had greater variance in the local Ca^2+^ signal decay indicating higher dyssynchrony in the local uptake of Ca^2+^ (*Figure 6E*). The amplitudes of the locCaTs correlated with the amplitude of the whole cell CaT (globalCaT) in all groups (Supplementary material online, *Figure* *S5B*). However, the dyssynchrony of the locCaT in RT and decay reduced drastically the globalCaT-amplitude/localCaT-amplitude ratio (synchrony efficiency) of female KO myocytes and also male KO myocytes (Supplementary material online, *Figure* *S5B*) despite the lower degree of dyssynchrony (*Figure 6D and E*). This indicates that the local dyssynchrony is a contributing factor in the PGC-1α deficiency-induced suppression of CaT amplitude (*Figure 5B*).

When we repeated this analysis in temporal domain (temporal-σ) over several CaTs (*Figure 6F, H, and I*), female KO cardiomyocytes showed the highest variance in both RT and decay of the local calcium signals among all the groups (*Figure 6H and I*). Dyssynchrony of the calcium signals may arise from transient changes in the local calcium release but also from irreversible structural changes like t-tubular disarray.^46^ Latter types of changes induce compromised calcium release and uptake at fixed spots in the cell substructures, whereas transient modifications are likely to contribute to beat-to-beat deviation. To distinguish these from each other, we divided the original line-scans into 2000 ms long frames (*Figure 6G*). After elimination of the beat-to-beat variance by averaging the frames, the remaining variance (structural-σ) was considered to originate from long lasting, structural modifications. This analysis revealed that part of the dyssynchrony characteristic to female KO cardiomyocytes results from the structural changes (*Figure 6J and K*) or changes that over last the beat-to-beat variation in local Ca^2+^ release and uptake. Collectively our data indicate that the single most important physiological difference between male and female PGC-1α-KO cardiomyocytes is the dyssynchronized local calcium release and uptake, which is likely to explain more severe cardiac phenotype and worse survival of female Heart-PGC-1α-KO mice.

### 3.7 PGC-1α deficiency leads to disruption of the t-tubular structures of cardiomyocytes

**Figure 7:**
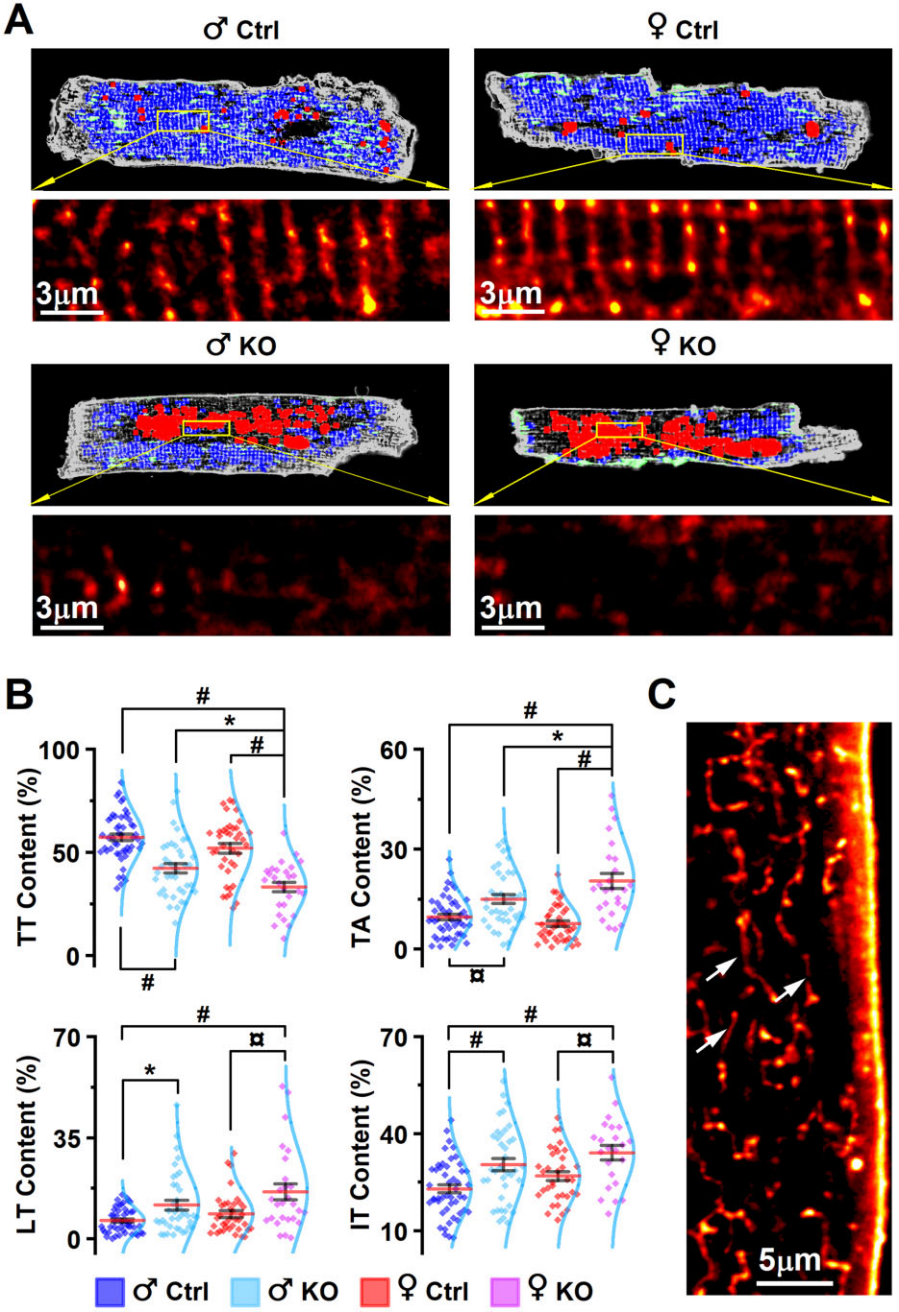
PGC-1α deficiency induces disruption of the cardiomyocyte T-tubule structures. (*A*) Representative confocal images of CellMask Orange Plasma Membrane labelled cardiomyocytes at the age of 22 weeks showing regular t-tubule structure (blue area), absence of t-tubules (red), longitudinal tubules (green), and non-regular tubules (grey) with enlargements below (yellow box and arrows). (*B*) Quantification of t-tubule structures [TT content—percentage of t-tubule regular structure, TA content—absence of t-tubules, LT content—longitudinal tubules, and IT content—irregular tubules; male Ctrl *n* = 3/50 (animals/cells), male KO *n* = 3/39, female Ctrl *n* = 3/39, female KO *n* = 3/27]. (*C*) Representative female KO cell shows longitudinal (arrows) and irregular tubule structures. Hierarchical linear model significance: *P < 0.05, ^¤^*P* < 0.01, ^#^*P* < 0.001.

As described, PGC-1α deficiency results in drastic changes of the global and subcellular calcium signalling of especially female cardiomyocytes. To further study the structural changes associated with it we next visualized t-tubule structures of the cardiomyocytes isolated from 22-week-old mouse ventricles. Indeed, the cardiomyocytes from Heart-PGC-1α-KO mouse ventricles showed distorted tubular system characterized by reduced cytosolic area with intact t-tubule (TT content) and increased area devoid of t-tubule (TA content; *Figure 7A and B*). As expected based on calcium dyssynchrony results, these changes were more pronounced in female KO myocytes (*Figure 7B*). Additionally, together with TT content and TA content changes, we found enhanced longitudinal t-system (LT content) in the myocytes of both male and female PGC-1α KO mice, which is a typical observation in HF.^51^ Similarly, irregular t-tubule structures were found in both male and female PGC-1α KO cardiomyocytes (*Figure 7B and C*).

Overall, the observed disruptive changes in t-tubule structures are sufficient to explain the dyssynchrony of the subcellular calcium signals in female PGC-1α KO (*Figure 6*). Male Heart-PGC-1α-KO had moderate changes in their subcellular calcium signals compared to females, mainly a decrease in the calcium release synchrony efficiency in 18-week-old animals (Supplementary material online, *Figure* *S5B*). However, at the age of 22 weeks male Heart-PGC-1α-KO cardiomyocytes showed similar, although milder changes in t-tubule structures, suggesting that the pathological development associated with PGC-1α deficiency eventually affects both sexes, although male mice develop HF later than females.

## 4. Discussion

The widely recognized importance of biological sex on cardiac physiology and pathogenesis has prompted specific recommendations for clinical and basic research.^2^ However, sex-specific clinical practices or therapies are emerging slowly in the absence of deeper understanding of the specific mechanism behind sex dimorphism in cardiac disease progression. Here, we have tested the hypothesis that energy metabolism has a central role in sex dimorphism of HF progression and that a signalling cascade involving PGC-1α might have a role in it. We demonstrate that mice deficient in cardiac PGC-1α expression develop dilated HF associated with suppression of energy metabolism, compromised calcium handling of cardiomyocytes, remodelling of electrophysiological properties of cardiomyocytes as well as substantial remodelling of gene expression. These changes occur in both sexes, but female mice develop a more severe structural and functional phenotype at a younger age. The HF endpoint of both sexes results from metabolic alterations associated with disruption of t-tubules and dyssynchrony of local calcium signals resulting in compromised calcium regulation of the cardiomyocytes.

Reduction of PGC-1α expression has been associated with the development of cardiac hypertrophy and HF,^52^^,^^53^ while increased PGC-1α expression has been suggested to mediate part of the effects of exercise.^54^ Supporting this, PGC-1α deletion compromises cardiac function and metabolism^27^^,^^55^ while moderate PGC-1α1 overexpression in the mouse heart induces beneficial effects on cardiac function^33^ and reduces ageing associated heart remodelling.^56^ Importantly, all these previous studies have been done by using only male animals, and possible sex-specific effects of PGC-1α have remained unknown. Our data, showing that female mice are more prone to develop HF resulting from PGC-1α deletion, support the idea that PGC-1α/PPAR and their target genes might be involved in sex disparity of cardiac energy metabolism and pathogenesis.^8^^,^^24^^,^^25^ The PGC-1α deficiency induces decline in all central aspects of cardiomyocyte energy metabolism, including substrate utilization, glycolysis, and oxidative phosphorylation (*Figure 3*), leading to compromised energy production common to advanced HF.^57^ Facing the compromised energy metabolism, the hearts of PGC-1α KO animals develop cardiac dilatation without compensatory hypertrophy at any stage before eventual HF. This metabolic suppression occurs similarly in both sexes, but why does it lead to more rapid and drastic contractile dysfunction and earlier death in female mice?

Compared to male hearts, female hearts have several properties that protect them against pathological developments. Female hearts develop more favourable forms of hypertrophy in response to pathological load^15^ associated with beneficial adaptation of cardiac energy metabolism^14^ compared to males. Our data indicate that when the glycolytic and oxidative metabolism are blunted by PGC-1α deficiency (*Figure 3B*), female cardiomyocytes suffer from more severe energy crisis compared to males (*Figure 3E*). This compromised energy metabolism is associated with faster progression of structural and functional HF in females compared to males, suggesting that maintenance of normal EC-coupling phenotype is more reliant on intact energy metabolism in female than male hearts. Furthermore, the effects of the PGC-1α deficiency indicate that energy metabolism-associated pathways mediate the female-specific beneficial adaptation against HF.

It is conceivable that the explanations for sex disparities in disease progression arise from pre-existing sex-related genetic and physiological differences that evoke sex-specific responses to pathological stress.^2^^,^^11^ As an example of these differences, central parameters of heart function, EF, and LV volume were different between sexes and females had smaller cardiac myocytes and hearts (*Figure 1*).^43^^,^^44^ At cellular level, female cardiomyocytes had longer APs (*Figure 4B*) and higher basal glycolysis rate compared to males (*Figure 3*). While PGC-1α deficiency equalized some of these functional differences between sexes such as AP length and energy metabolism, it increased the sex disparity in central measures of LV function and Ca^2+^ signalling.

At the level of transcription, the differences between 18-week-old male and female control hearts were small and lack of PGC-1α did not lead to considerable changes (*Figure 2B and C*), even though sex disparity in transcriptomic response to knockout seemed evident (*Figure 2E*). The explanation for this is that the great majority of genes altered significantly only in either of the sexes had a similar trend in the opposite sex (Supplementary material online, *Figure* *S2D* *and* *E*). This implies that the strength of the knockout effect on specific genes is different, which is likely reflecting the different functional outcome between males and females. Even at the age of 22 weeks, just before the decline in female KO survival, transcripts related to HF (*Figure 1H*), PGC-1α pathway (*Figure 2G–J*), electrophysiology or Ca^2+^ signalling (Supplementary material online, *Figure* *S3*) showed only a few sex-specific changes. Overall, the transcriptomic response to PGC-1α deficiency is very similar between sexes, further suggesting that it is the pre-existing differences in physiological traits of males and females that is the main reason for faster progression on phenotype in female PGC-1α KO.

Heart-PGC-1α-KO mice demonstrate the intimate interplay between compromised energy metabolism and consequent development of HF. During this process, hearts are prone to develop contractile dysfunction at the organ and cellular level originating from disruption of cardiomyocyte t-tubules and loss of coordination of the cellular Ca^2+^ handling. In healthy adult ventricular cardiomyocytes, efficient Ca^2+^ release and uptake rely on highly organized coupling between plasma membrane LTCCs and SR RyRs brought together by dense arrays of t-tubules.^58^ The fundamental purpose of these structures is to reduce the delay between electrical excitation and Ca^2+^ release as well as reduce the Ca^2+^ diffusion distances within the cytosol.^59^ Because of this essential role in controlling the local Ca^2+^ release, disruptions of these structures effectively compromise the whole cell Ca^2+^ signals as well as contraction. Compromised coupling between LTCCs and RyRs as well as dysfunction of CRUs can result in dyssynchrony of the local Ca^2+^ release and uptake at spatial (dyssynchrony) and temporal (alternans) domain.^47–49^^,^^60^ During the development of HF, the substructures of cardiomyocytes, including the t-tubules, revert to an immature phenotype,^51^ which is likely a major contributing factor to the compromised calcium signalling in HF and a predictive factor for LV function.^61^

According to our data, the major difference between female and male Heart-PGC-1α-KO hearts is the earlier appearance of the dyssynchrony of the local Ca^2+^ signals of the female KO cardiomyocytes, associated with parallel degradation of the t-tubules. While specific molecular mechanisms for this subcellular remodelling have not yet been identified, proteins such as BIN1 and junctophilin-2 have been implicated in the regulation of t-tubule development and pathological remodelling.^62^ Similarly, specific signals or signalling cascades for pathological t-tubule remodelling have not been identified, but it has been shown that cardiac workload and ventricular wall stretch regulate the t-tubule structures.^63^^,^^64^ Compared to the commonly used HF models demonstrating t-tubule remodelling Heart-PGC-1α KO is different in many ways, since the initial pathological stimuli does not include workload or ventricular wall stretch, but instead the primary trigger is the suppression of the energy metabolism. Accordingly, at the level of transcripts (*Figure 2*) PGC-1α deficiency affects predominantly genes involved in energy metabolism with very little effect on gene patterns typically involved in workload or wall stretch-induced HF or t-tubule remodelling. In contrast, according to metabolomic analysis the metabolite profile of the Heart-PGC-1α KO ventricles^27^ as well as loaded or infracted hearts^65^ show significant similarities in changes of energy metabolites and degradation products of membrane lipids. Considering the high turnover rate of t-tubules^66^ one could speculate that the maintenance of the complex t-tubular arrangements is an energy demanding process, and hence compromised energy production might affect t-tubular network directly. While more specific pathways explaining the t-tubule changes in HF may hopefully emerge, based on our current data it could be hypothesized that independently of the cause, compromised energy metabolism is associated with membrane degradation affecting t-tubules.

To summarize, this study demonstrates that the integrity of the subcellular Ca^2+^ release and uptake machinery is dependent on energy metabolism and suggests that female hearts are more prone to suffer from contractile dysfunction in conditions with compromised production of cellular energy. Furthermore, our data suggest that PGC-1α has a central role in sex disparity of metabolic cardiomyopathy.

## Supplementary material

Supplementary material is available at *Cardiovascular Research* online.

## Authors’ contributions

Conception: P.T. Design of the experiments, collection, analysis, and interpretation of data: N.N., M.M., T.T., L.H., and P.T. Providing the materials: J.L.R. Drafting the article and reviewing: M.M., N.N., T.T., L.H., J.L.R., and P.T. All authors approved the definitive version of the manuscript.

**Conflict of** **interest:** The authors declare no conflicts of interests.

## Funding

This work was supported by the Academy of Finland [325510 to P.T.] and Sigrid Juselius Foundation (P.T.).

## Data availability

The data that support the findings of this study are available from the corresponding author upon reasonable request.

## Supplementary Material

cvab188_Supplementary_Data

## Notes

Translational perspective Biological sex is an important variable in clinical medicine, cardiac physiology, and pathogenesis. However, sex-specific clinical practices or therapies are emerging slowly in the absence of deeper understanding of the specific mechanism behind sex dimorphism in cardiac disease progression. Here, we show that energy metabolism has a central role in sex dimorphism of heart failure (HF) progression and that a signalling cascade involving PGC-1α might have a role in it. We provide insights into sex-specific mechanisms of HF development which are necessary to identify sex-specific treatment practices for cardiovascular diseases.
