# Supplementary material for: PGC-1α deficiency reveals sex-specific links between cardiac energy metabolism and EC-coupling during development of heart failure in mice
Source: Cardiovasc Res. 2021 Jun 4;118(6):1520–34. doi: 10.1093/cvr/cvab188 (PMC9074965; doi:10.1093/cvr/cvab188)
Supplement: cvab188_Supplementary_Data [file cvab188_supplementary_data.docx]

**Supplementary material**

**PGC-1α-deficiency reveals sex specific links between cardiac energy metabolism and EC-coupling during development of heart failure in mice:** *Nikolay Naumenko, Maija Mutikainen, Lari Holappa,* *Jorge L. Ruas, Tomi Tuomainen and Pasi Tavi*

# **Supplementary figures**





**Supplementary Figure S1.** Morphology of control and KO ventricles assessed with MTC staining from 12- and 18-week-old male and female mice. Inset (square) of each figure is shown on the right side.





**Supplementary Figure S2. RNA sequencing analysis. A**, Histogram showing counts of log_2_ transformed average FPKM values from all genes present in the employed Ensembl gene annotation set. Genes with low expression were filtered out of the analysis by choosing only genes that had at least two samples above chosen cut-off (log_2_(FPKM) > 0, red dashed line) in at least one of the four experimental groups. Final number of genes in the subsequent analyses was 11970. **B**, Principal component analysis on the expression levels of filtered genes showing clustering of the samples according to experimental groups. **C**, Heatmaps showing log_2_ transformed fold change of genes that were differentially expressed between males and females only in controls (*left*), both in controls and knockouts (*middle*) and only in knockouts (*right*). **D-G**, Comparison of PGC1-α knockout induced expression changes between sexes (*left*) and heatmaps showing logarithmic expression level of genes in all samples (*right*) in chosen gene sets. Hierarchical clustering in the heatmaps was applied for rows (genes) and columns (samples). In left panel of **D** and **F** genes are ordered according to ascending fold change in males and female fold change (red) is overlaid on male fold change (blue). In left panel of **E** and **G** genes are ordered according to ascending fold change in females and male fold change is overlaid on female fold change (3 animals in each group, 18-week-old).





**Supplementary Figure S3.** **Ventricular gene expression in 18- and 22-week-old male and female mice.** **A**, Expression of sodium channel α-subunit (*Scn5a*), α-subunit calcium channel (*Cacna1c*), NCX (*Slc8a1*), sodium/potassium ATPase (*Atp1a1*) (upper panel) and selected potassium channel subunits (lower panel, refer to Suppl. Table S1 for gene abbreviations). **B**, Expression of calcium handling related genes ryanodine receptor (*Ryr2*), SERCA2 (*Atp2a2*), calsequestrin 2 (*Casq2*) and phospholamban (*Pln*), and ratio of *Atp2a2* to *Pln*. For 18-week-old mice n = 8 and for 22-week-old mice n = 5. In Kcna5, Kcnd2, Kcna4, Kcnj2, Kcne1 and Kcnb1 18-week-old male control and knockout n = 7. * P<0.05, ¤ P<0.01 and # P<0.001.





**Supplementary Figure S4. Calcium transient time course in male and female PGC-1α knockout. A**, Reconstruction of CaT shape: rise time (left) and decay time (right) (Note: different time scale for left and right panels). **B**, Means of rise time (left), maximum rise (middle) and decay (right panel) slopes. Plot details: mean is red line, whisker is SEM, cyan line is distribution (male Ctrl n = 89, male KO n = 88, female Ctrl n = 47 and female KO n = 56, at least 3 animals in each group, 18-week-old). * P<0.05, ¤ P<0.01 and # P<0.001.





**Supplementary Figure S5. Variation of locCaT amplitudes** **in PGC-1α knockout. A**, Means of standard deviation of locCaT amplitudes (Amplitude-σ, left). Right panel: correlation between CaT amplitude-σ and SR Ca^2+^-content. **B**, Synchrony efficiency index in PGC-1α KO cardiomyocytes. Left panel: correlation between mean of locCaT amplitudes and global CaT amplitude shows dyssynchrony of calcium release in all groups (slopes of the linear fitting are more than 1). Right panel: synchrony efficiency index was assessed as ratio of mean of locCaTs and global CaT amplitudes. Plot details: red horizontal line is mean, whisker is SEM, and cyan line is distribution (male Ctrl n=89, male KO n=88, female Ctrl n=47 and female KO n=56, at least 3 animals in each group, 18-week-old). * P<0.05, ¤ P<0.01 and # P<0.001.

**Supplementary Figure S6. Original western blot images. A,** PGC-1α blot shown in Figure 1A (left) and corresponding multichannel image to show molecular weight markers together with sample signals (right). **B,** Lamin B blot shown in Figure 1A. **C,** Upper AMPK blot shown in Figure 3D (left) and ponceau S staining from the same blot (right). **D,** Lower AMPK blot shown in Figure 3D (left) and ponceau S staining from the same blot (right). **E,** Upper p-AMPK blot shown in Figure 3E (left) and ponceau S staining from the same blot (right). **F,** Lower p-AMPK blot shown in Figure 3E (left) and ponceau S staining from the same blot (right). All sample lanes from each blot are shown in the corresponding cropped images in the main text figures.

# **Supplementary Table S1. Nucleotide sequences of primers and probes used in quantitative RT-PCR analysis.**

| Short name | Name, Gene ID |  | Nucleotide sequence |
| --- | --- | --- | --- |
| Hprt | hypoxanthine phosphoribosyltransferase | 5' | GAACCAGGTTATGACCTA |
|  |  | 3' | TCTCCTTCATGACATCTC |
|  | 15452 | probe | TTCAGTCCTGTCCATAATCAGTCCAT |
| Ppargc1a | peroxisome proliferator activated receptor, | 5' | AGCGACCAATCGGAAATCAT |
|  | gamma,coactivator 1 alpha | 3' | GCAAGTTTGCCTCATTCTCTTCA |
|  | 19017 | probe | TCCAACCAGTACAACAATGAGCCTGCG |
| Ppargc1b | peroxisome proliferative activated receptor, | 5' | GGACGAGCTTTCACTGCTACAGA |
|  | gamma, coactivator 1 beta | 3' | TCCTTCAGAGCGTCAGAGCTT |
|  | 170826 | probe | CTCCTCCTGGCCACATCCTCCCC |
| Ppara | peroxisome proliferator activated receptor | 5' | ACGATGCTGTCCTCCTTGATG |
|  | alpha | 3' | GTGTGATAAAGCCATTGCCGT |
|  | 19031 | probe | ACAAAGACGGGATGCTGATCGCG |
| Pparg | peroxisome proliferator activated receptor | 5' | AGTGGAGACCGCCCAGG |
|  | gamma | 3' | GCAGCAGGTTGTCTTGGATGT |
|  | 19016 | probe | TTGCTGAACGTGAAGCCCATCGAG |
| Esrra | estrogen related receptor, alpha | 5' | GATCGAGAGATAGTGGTC |
|  |  | 3' | CTCTGCAGTACTGACATC |
|  | 26379 | probe | CATCCCAGGCTTCTCCTCACT |
| Esrrb | estrogen related receptor, beta | 5' | GCCACCAATGAATGTGAG |
|  |  | 3' | GCTGTTCTCCGAATCCAG |
|  | 26380 | probe | TTGTACTTCTGGCGGCCTCC |
| Esrrg | estrogen-related receptor gamma | 5' | CCACGAATGAATGTGAGA |
|  |  | 3' | CTCAGCATCTATTCTGCG |
|  | 26381 | probe | TACTTCTGCCGACCTCCACG |
| Mef2c | myocyte enhancer factor 2C | 5' | GGAACACGCCTGTCACCTAAC |
|  |  | 3' | ATGAGTGCCATACGCCAATG |
|  | 17260 | probe | AGCACGCTCACAAACCTGCAGGC |
| Bdh1 | 3-hydroxybutyrate dehydrogenase, type 1 | 5' | AAGGCTTCCTTGTATTTG |
|  |  | 3' | CCTTCTCAGGATCTTTCA |
|  | 71911 | probe | ATCGTCTCCACCGCCTTCTC |
| Sod2 | superoxide dismutase 2, mitochondrial | 5' | GAGATGTTACAACTCAGG |
|  |  | 3' | GCTTCTCCTTAAACTTCTC |
|  | 20656 | probe | TGATAGCCTCCAGCAACTCTCC |
| Tfam | transcription factor A, mitochondrial | 5' | TTCGTTACGACAATGAAATGAAGTC |
|  |  | 3' | TCGACGGATGAGATCACTTCG |
|  | 21780 | probe | TGGGAAGAGCAGATGGCTGAAGTTGG |
| mt-Cytb | cytochrome b, mitochondrial | 5' | GACAACTACATACCAGCTAATCCACTAAA |
|  |  | 3' | GAATGGCGTATGCAAATAGGAAA |
|  | 17711 | probe | ACCCCACCCCATATTAAACCCGAATGA |
| Myh6 | myosin, heavy polypeptide 6, cardiac muscle, | 5' | GGTGCCAAGAAGATGCACG |
|  | alpha | 3' | TTATGTTTATTGTGTATTGGCCACAG |
|  | 17888 | probe | CGAGGAATAACCTCTCCAGCAGACCCTC |
| Myh7 | myosin, heavy polypeptide 7, cardiac muscle, | 5' | AGCTCTAAGGGTGCCCGTG |
|  | beta | 3' | TGCTTCCACCTAAAGGGCTG |
|  | 140781 | probe | AGCCCTCAGACCTGGAGCCTTTGC |
| Acta1 | actin, alpha 1, skeletal muscle | 5' | TCCTCCGCCGTTGGCT |
|  |  | 3' | AATCTATGTACACGTCAAAAA |
|  | 11459 | probe | CATCGCCGCCACTGCAGCC |
| Nppa | natriuretic peptide type A | 5' | GAAAAGCAAACTGAGGGCTCTG |
|  |  | 3' | CCTACCCCCGAAGCAGCT |
|  | 230899 | probe | TCGCTGGCCCTCGGAGCCT |
| Nppb | natriuretic peptide type B | 5' | AGGCGAGACAAGGGAGAACA |
|  |  | 3' | GGAGATCCATGCCGCAGA |
|  | 18158 | probe | CATCATTGCCTGGCCCATCGC |
| Scn5a | sodium channel, voltage-gated, type V, alpha | 5' | CAGCAGCTTCCGTAGGTTCAC |
|  |  | 3' | TTTCAGCCATGCGCTTCTC |
|  | 20271 | probe | TGGCCGCCAGTGACTCCCG |
| Cacna1c | calcium channel, voltage-dependent, L type, | 5' | TTGACAATGTTTTGGCAGCC |
|  | alpha 1C subunit | 3' | TCTGGCCACCCTTCGA |
|  | 12288 | probe | TGATGGCTCTCTTCACCGTCTCCACC |
| Slc8a1 | solute carrier family 8 (sodium/calcium | 5' | TTGTTTTCCCATGTTGACCATATAA |
|  | exchanger), member 1 | 3' | GAGCCAGTACATTCAGTGGTTTCA |
|  | 20541 | probe | TGCAGATACAGAGGCAGAAACAGGAGGAA |
| Atp1a1 | ATPase, Na+/K+ transporting, | 5' | TGCTCTCTTCTCTTTCTAGTC |
|  | alpha 1 polypeptide | 3' | GTCACCATGCTCCGATAC |
|  | 11928 | probe | CAGGCTCATACTTGTCTCGTCCA |
| Kcna5 | potassium voltage-gated channel, | 5' | GAGTCATCCTCTTCTCCA |
|  | shaker-related subfamily, member 5 | 3' | TAGCCTACAGTGGTCATA |
|  | 16493 | probe | TACTTCGCAGAGGCAGACAATCA |
| Kcnd2 | potassium voltage-gated channel, | 5' | CCTTCTGGTACACCATCG |
|  | Shal-related family, member 2 | 3' | GACTGAAGTTCGACACGA |
|  | 16508 | probe | CGCAATGACCAAGACTCCGC |
| Kcna4 | potassium voltage-gated channel, | 5' | GTGGAAAAGGGGAAACAA |
|  | shaker-related subfamily, member 4 | 3' | CAGGAAATGAAGAGCATCC |
|  | 16492 | probe | ATTCACAAGAAGCACTTCACCATTCC |
| Kcnj2 | potassium inwardly-rectifying channel, | 5' | CCACTTCCACTCCATGTC |
|  | subfamily J, member 2 | 3' | GATGGATGCTTCCGAGAA |
|  | 16518 | probe | ACCAGCAACAGGACAAGTTCTCT |
| Kcne1 | potassium voltage-gated channel, | 5' | CTGCCACACACCTTCCTGAA |
|  | Isk-related subfamily, member 1 | 3' | AAAGATCCGCTTGTCACCTGTAG |
|  | 16509 | probe | TGAAGCCATTGTCGTGAACCCCACA |
| Kcnb1 | potassium voltage gated channel, | 5' | CTTGGTATTTTGCTGTTTTC |
|  | Shab-related subfamily, member 1 | 3' | GCTCCTGTTTTGAGAAAG |
|  | 16500 | probe | ACTCTGCCTCACCATCCGTC |
| Ryr2 | ryanodine receptor 2, cardiac | 5' | CAGCAGCCCCCACAGG |
|  |  | 3' | TTCCATGTAGCCGCTGCTC |
|  | 20191 | probe | TCATTGCGGTTCACTATGTCCTGGAGG |
| Atp2a2 | ATPase, Ca++ transporting, cardiac muscle, | 5' | CAGCCATGGAGAACGCTCA |
|  | slow twitch 2 | 3' | TCGTTGACCCCGAAGTGG |
|  | 11938 | probe | ACAAAGACCGTGGAGGAGGTGCTGG |
| Casq2 | calsequestrin 2 | 5' | GGAACATCAAAGACCCACCCT |
|  |  | 3' | TCGTCTTCCCATGTTTCAAACA |
|  | 12373 | probe | CGTCGCTTGCGCCCAGAGG |
| Pln | phospholamban | 5' | CAGGAGAGCCTCCACTATTGAAA |
|  |  | 3' | GATGAGGCAGAAATTGATAAATAGGTT |
|  | 18821 | probe | CCTCAGCAAGCACGTCAGAATCTCCA |

# **Supplementary Table S2. Top 20 Gene Ontology Biological process terms (uncorrected p<0.1) from enrichment analysis of differentially expressed genes between experimental groups**

| **Biological process term** | **Bonferroni corrected p-value** |
| --- | --- |
| **female Ctrl vs male Ctrl** |  |
| translational initiation | 1.76E-01 |
| regulation of gene expression | 3.09E-01 |
| ossification | 9.69E-01 |
| histone H3-K27 demethylation | 9.85E-01 |
| oxidation-reduction process | 9.94E-01 |
| histone H3-K4 demethylation | 9.97E-01 |
| extracellular matrix organization | 1.00E+00 |
| immune response | 1.00E+00 |
| covalent chromatin modification | 1.00E+00 |
| formation of translation preinitiation complex | 1.00E+00 |
| collagen fibril organization | 1.00E+00 |
| extrinsic apoptotic signaling pathway via death domain receptors | 1.00E+00 |
| cerebellum development | 1.00E+00 |
| embryonic organ development | 1.00E+00 |
| **female KO vs male KO** |  |
| heart development | 9.97E-01 |
| cartilage development | 9.98E-01 |
| histone H3-K27 demethylation | 1.00E+00 |
| oxidation-reduction process | 1.00E+00 |
| translational initiation | 1.00E+00 |
| histone H3-K4 demethylation | 1.00E+00 |
| regulation of gene expression | 1.00E+00 |
| lung development | 1.00E+00 |
| immune response | 1.00E+00 |
| cell-cell junction organization | 1.00E+00 |
| signal transduction | 1.00E+00 |
| **male KO vs male Ctrl** |  |
| oxidation-reduction process | 2.18E-17 |
| G-protein coupled receptor signaling pathway | 4.05E-08 |
| metabolic process | 2.25E-07 |
| immune response | 8.86E-06 |
| inflammatory response | 4.36E-05 |
| antigen processing and presentation of exogenous peptide antigen via MHC class II | 6.59E-04 |
| fatty acid metabolic process | 7.37E-04 |
| neutrophil chemotaxis | 1.68E-03 |
| cell adhesion | 1.74E-03 |
| ion transport | 2.04E-03 |
| lipid metabolic process | 2.25E-03 |
| signal transduction | 5.75E-03 |
| collagen fibril organization | 6.27E-03 |
| cellular response to interleukin-1 | 1.42E-02 |
| respiratory electron transport chain | 1.53E-02 |
| immune system process | 3.79E-02 |
| monocyte chemotaxis | 7.65E-02 |
| chemokine-mediated signaling pathway | 7.65E-02 |
| positive regulation of ERK1 and ERK2 cascade | 8.10E-02 |
| biosynthetic process | 1.70E-01 |
| **female KO vs female Ctrl** |  |
| oxidation-reduction process | 1.74E-09 |
| metabolic process | 3.75E-08 |
| fatty acid metabolic process | 1.04E-03 |
| cell adhesion | 2.74E-03 |
| tricarboxylic acid cycle | 4.03E-03 |
| lipid metabolic process | 6.66E-03 |
| regulation of muscle contraction | 5.60E-02 |
| fatty acid beta-oxidation | 9.97E-02 |
| fatty acid beta-oxidation using acyl-CoA dehydrogenase | 1.04E-01 |
| G-protein coupled receptor signaling pathway | 1.30E-01 |
| collagen fibril organization | 1.83E-01 |
| 2-oxoglutarate metabolic process | 4.13E-01 |
| ion transport | 4.80E-01 |
| cell surface receptor signaling pathway | 5.56E-01 |
| positive regulation of cell adhesion mediated by integrin | 6.59E-01 |
| aging | 8.02E-01 |
| cell-cell signaling | 8.99E-01 |
| response to cold | 8.99E-01 |
| extracellular matrix organization | 9.71E-01 |
| lipid homeostasis | 9.86E-01 |

# **Supplementary Table S3. Action potential parameters (Mean ± SEM).**

| Gender | Genotype | N | Amplitude, mV | Resting membrane potential, mV | Maximum rise slope, mV/ms |
| --- | --- | --- | --- | --- | --- |
| Male | Ctrl | 8/26 | 126.5 ± 1.0 | -77.3 ± 0.6 | 427.4 ± 11.7 |
|  | KO | 7/24 | 124.1 ± 1.1 | -76.0 ± 0.5 | 388.0 ± 12.4 |
| Female | Ctrl | 5/22 | 126.3 ± 1.4 | -76.9 ± 0.8 | 393.3 ± 13.6 |
|  | KO | 5/20 | 123.4 ± 2.1 | -77.1 ± 1.1 | 364.4 ± 13.9 |

# **Supplementary Table S4. Holding currents**

| Gender | Genotype | I_CaL_, N | I_CaL_, holding current, pA | I_K_, N | I_K_, holding current, pA |
| --- | --- | --- | --- | --- | --- |
| Male | Ctrl | 3/16 | -170.4 ± 18.6 | 8/30 | -444.0 ± 54.8 |
|  | KO | 3/11 | -134.6 ± 29.4 | 7/32 | -333.7 ± 39.1 |
| Female | Ctrl | 3/16 | -161.9 ± 29.5 | 5/10 | -332.1 ± 94.7 |
|  | KO | 3/8 | -173.2 ± 52.9 | 4/17 | -345.5 ± 89.0 |

# **Supplementary methods**

## **Ethical approval**

All animal experiments were carried out with an authorization by The National Animal Experiment Board of Finland (animal experimentation permit number ESAVI/7867/2018) and following the guidelines of The Finnish Act on Animal Experimentation, which comply with the guidelines from Directive 2010/63/EU of the European Parliament on the protection of animals*.*

## **Experimental animals**

The homozygous cardiac spesific PGC-1α KO (briefly as Heart-PGC-1α KO in the text) male and female mice were achieved by cross-breeding floxed *Pgc-1α* (B6‐Ppargc1a‐flox/J/KI) with hemizygous Myh6-Cre mice (B6.FVB-Tg(Myh6-cre)2182Mds/J, JAX stock #011038) ^1^. Age matched littermate controls were homozygous for floxed *Pgc-1α* and negative for Myh6-Cre. Tissue samples were collected at the ages of 12, 18, 20, 21 and 22 weeks. For age-follow up, mice were aged until 250 days. The animals were reared and kept in standard animal housing conditions in the National Laboratory Animal Center of the University of Eastern Finland in Kuopio. All animal experiments were carried out in the daytime (from 8 a.m. to 3 p.m.) and animals were fed *ad libitum*. For tissue collection, animals were euthanized with CO_2_ inhalation followed by cervical dislocation. For cell isolation, animals were euthanized by cervical dislocation without preceding CO_2_ inhalation.

## **Survival follow-up**

Heart-PGC-1α-KO mice were aged as long as they did not show any signs of health problems. Special attention was paid to the cardiac function by echocardiography and overall health monitoring. Any signs of impaired cardiac function, such as shortness of breath or inactivity, were considered as time points for euthanasia according to guidelines and specifications of animal experimentation permit (ESAVI/7867/2018).

## **Echocardiography**

A high-resolution Vevo2100 Ultrasound imaging system (VisualSonics Inc., Toronto, Canada) with a 30 MHz operating frequency MicroScan transducer was used for echocardiography. The animals were imaged under inhalation anaesthesia [induction with 3.5-4% isoflurane (Baxter International Inc., Deerfield, IL, USA) and 350-400 mL/min air, maintenance with 2-2.5% isoflurane and 200-250 mL/min air] and kept on a heated platform during and shortly after the imaging. Ventricular dimensions and functional parameters, including left ventricular volume (LV_vol_), anterior- (LVAW) and posterior wall thicknesses (LVPW) in diastole and systole, ejection fraction (EF), heart rate (HR) and cardiac output (CO) were determined from short-axis M-Mode measurements from 10-, 12- and 16-weeks-old animals.

## **RNA sequencing**

Total RNA from left ventricular tissue of 18-week-old mice was isolated with TRI reagent (Sigma-Aldrich) using TissueLyzer II (Qiagen) in homogenization. Library preparation and sequencing were performed in the Finnish Functional Genomics Centre (Turku, Finland). Quality of the isolated RNA was assessed with Fragment Analyzer (Advanced Analytical Technologies, Inc.) and RQN values of the samples varied between 8.7 and 10. RNA sequencing libraries were prepared from 300 ng total RNA according to Illumina TruSeq Stranded mRNA Sample Preparation Guide (#15031047). First, poly-A containing mRNA was purified with poly-T magnetic beads after which mRNA was fragmented using divalent cations in elevated temperature. After purification and PCR enrichment of the strand specific cDNA libraries, unique indexing adapters were ligated each sample to allow sequencing in same flow cell lane. Quality of the libraries was assessed with Fragment Analyzer and the average library fragment size was 250-350 bp. Libraries were sequenced with Illumina HiSeq 3000 instrument using 50 bp single-end sequencing. Base calling was done with Illumina’s *bcl2fastq2* software using automatic adapter trimming. Mapping of the sequencing reads stored in fastq files was performed with *hisat2* software ^2^ using indexes for mouse GRCm38 genome build. Mapped reads were annotated and quantified with *QoRTs* software package ^3^ using Ensembl’s annotation release 92 for GRCm38 genome. Deseq2 method ^4^ in *R* software environment was used to determine differential expression between experimental groups. In clustered heatmaps, created with *heatmap.2* function of gplots package in *R*, logarithmic transformed expression values acquired with *rlog* function of the Deseq2 package were used. Gene enrichment analysis for sets of differentially expressed genes was performed with web-based *DAVID* software ^5^.

## **Single cell isolation**

Adult cardiomyocytes from 18-week-old mice (±6 days) were isolated as described previously ^6^ (AfCS Procedure Protocol PP00000125: http://www.signaling-gateway.org). Animals from each experimental group at the same age were used in individual assays. Briefly, isolated hearts were placed in a Langendorff apparatus for perfusion (37°C, 3 mL/min) with a trypsin (Sigma) and liberase (Roche Applied Science) solution. After perfusion, ventricles were cut into small pieces and gently minced with a Pasteur pipette. Concentration of Ca^2+^ in solution was increased slowly up to 1 mmol/L. Freshly isolated cardiomyocytes were plated on laminin-coated coverslips for electrophysiological and Ca^2+^ imaging recordings or on Matrigel for energy metabolism analysis.

## **Analysis of energy metabolism in isolated cardiomyocytes**

Energy metabolism was assessed with Seahorse XF24 analyzer (Agilent Technologies) as described earlier ^1^. Isolated cells were plated on Matrigel (BD Biosciences) coated XF24 cell plates (Agilent Technologies) in XF Minimal Base Medium (Agilent Technologies) supplemented with 2 mmol/L GlutaMAX (ThermoFisher Scientific), 10 mmol/L butanedione monoxime (Sigma-Aldrich) and either 0.2 mmol/L palmitate (Sigma-Aldrich) conjugated to BSA (Sigma-Aldrich) or 4.5 g/L glucose. Glucose supplemented assay medium contained the same amount of BSA as palmitate medium. After basal measurements, 1.5 µmol/L carbonyl cyanide-4-(trifluoromethoxy) phenylhydrazone (FCCP) was added to determine maximal metabolic rates. Results were normalized to total cellular protein.

## **Ca^2+^-imaging of isolated cardiomyocytes**

For Ca^2+^-imaging experiments only rod-shaped cells with visible response to electrical stimulation were used. Cytosolic calcium signals were measured as described previously ^6^ using the confocal imaging system (FluoView 1000, Olympus, Japan) mounted on inverted Olympus IX81 microscope equipped with 60x water immersion objective. Cells were loaded with 7.5 µmol/L Fluo4 AM ester fluorescent Ca^2+^ indicator (Invitrogen, Eugene, OR) at 37C° in Dulbecco’s modified Eagle medium (DMEM) containing GlutaMAX (Invitrogen) in the incubator (5% CO_2_). After 20 min of incubation with Fluo4, the cells were placed to microscope recording chamber continuously perfused with preheated to 37C° DMEM supplemented with 1 mmol/L probenecid (pH 7.4, bubbled with 95% O_2_ and 5% CO_2_). The Fluo4-loaded cells were exited at 488 nm and the emission was collected from 500 to 600 nm. Line-scan mode was used. The temporal resolution was 2 µs per pixel with the frame resolution of 800*800 pixels. Calcium transients were elicited by electrical stimulation (0.5 – 5 Hz) through two platinum wires located on both sides of the chamber connected to stimulator (Grass Instruments Co., USA). Fluorescence intensity of Ca^2+^ signals is expressed as F/F_0_ ratio, where F is the background subtracted fluorescence intensity and F_0_ is the background subtracted minimum fluorescence value measured from each cell at rest. Images were analysed using FluoView 4.0 (Olympus, Japan) and ImageJ 1.5 (https://imagej.nih.gov/ij/).

## **Ca^2+^ flux protocol**

To estimate calcium fluxes in Fluo4-loaded cardiomyocytes the fast-local application (eight-channel manifold, 37C°, Cell MicroControls, USA) was used. To improve speed of compound exchange, the local suction tube was located on opposite side of local application manifold. To estimate calcium fluxes the cells were first perfused with normal Tyrode solution (NT; see graphical representation of protocol Fig. 5A) with continuous electrical stimulation at 0.5 Hz. At this step, we measured basic calcium transient characteristics: amplitude and decay duration (epoch a). Next, perfusion solution was changed to 0 Ca^2+^ and 0 Na^+^ solution (0Ca^2+^/0Na^+^) with tetracaine (TC, 20 sec, epoch b). The shift in baseline level considered to indicate the sarcoplasmic reticulum calcium leak ^7^ through ryanodine receptors (RyR). Next, a fast and short caffeine pulse was applied (1^st^ Caffeine, 2sec, epoch c) followed by a change back to Tyrode solution 0Ca^2+^/0Na^+^ Tyrode solution. Amplitude of caffeine transient was considered to reflect sarcoplasmic reticulum (SR) calcium content, and single exponential decay of caffeine transient SR Ca^2+^-ATPase (SERCA) activity (epoch d). After five seconds, we applied second extended caffeine pulse (2^nd^ Caffeine, for 30 sec). At the middle of second caffeine application 0 Ca^2+^/0Na^+^ solution was changed to NT resulting in two phasic caffeine transient decay. Slow caffeine pulse decay (epoch e), which depicts the plasma membrane Ca^2+^ ATPase (PMCA) activity, was fitted by linear function. Fast caffeine pulse decay (epoch f) reflects the sodium-calcium exchanger (NCX) activity, which was estimated by single exponential fitting function. Normal Tyrode solution contains (in mmol/L): 130 NaCl, 5.4 KCl, 1 CaCl_2_, 1 MgCl_2_, 0.3 Na_2_HPO_4_, 10 HEPES, and 5.5 glucose (pH 7.4 with NaOH). Sodium chloride in 0 Ca^2+^/0 Na^+^ Tyrode solution was equimolar replaced with *N*-methyl-glucamine and calcium chloride was removed.

## **Analysis of the spatio-temporal characteristics of calcium release**

For detailed assessment of spatial and temporal calcium signals, we analysed time course parameters of the local calcium transients (locCaT) at subcellular level. Pixel resolution for all calcium imaging was 0.397 µm, and fluorescent intensity was analysed for every single pixel of line-scans. Rise time (RT) and decay time 66% (D66) of locCaT were analysed. Earlier it has been shown that Ca^2+^ transients with greater dyssynchrony of Ca-release have higher variation in the time course of locCaTs within the cell ^8^. Consequently, the degree of dyssynchrony was assessed as the standard deviation. For spatial dyssynchrony characterization, only one calcium transient was taken into analysis from each individual cell. Obtained profiles of RT and D66 were used to calculate standard deviation which was considered as spatial dyssynchrony index of calcium release (spatial-σ; see graphical explanation Fig. 6AB). However, such kind of inhomogeneity in calcium release may consist of beat-to-beat deviation (temporal-σ) and persistently silent or absent release sites (structural-σ) ^9^. To measure beat-to-beat calcium release dyssynchrony (temporal-σ; see graphical explanation Fig. 6D) five calcium transients were analyzed ^8^. The values of RT and D66 of locCaTs were taken to calculate standard deviation and mean of standard deviations for given cell was considered as temporal-σ. Disarray in the local calcium release resulting from structural changes such as t-tubule remodeling is consistent between beats ^9^. Thus, in order to evaluate structural -σ, first we eliminated beat-to-beat deviation in calcium release by averaging five frames (Fig. 6E, duration is 2000 ms) of original line-scan with five consecutive calcium transients as described previously ^10^. Resulting averaged line-scan frame was analyzed the similar way as for spatial-σ.

## **Whole cell patch-clamp**

Coverslips with attached cells were transferred to the recording chamber of microscope and perfused with preheated DMEM solution (37C°, pH=7.4, bubbled with 95% O_2_ and 5% CO_2_). For action potentials (APs) recordings patch-clamp amplifier Axopatch 200B in combination with a Digidata 1440A and Clampex 10 software (Molecular Devices Inc., Sunnyvale, CA, USA) were used as described previously ^6^. The Ag/AgCl half-cell electrode (World Precision Instruments Inc., Sarasota, FL, USA) connected to the bath via an agar bridge was used as ground electrode. Patch pipettes were pulled from borosilicate glass capillary tubing (ID 0.86 mm, Harvard Apparatus, Edenbridge, UK) with a micropipette puller (Sutter P-97, Sutter Instrument Company, Novato, CA, USA) and fire polished. Patch pipette resistances were 4–6 MΩ when filled with the pipette solution containing (in mmol/L): K-aspartate 120, KCl 8, NaCl 7, MgCl_2_ 1, Na_2_-phosphocreatine 2, Mg-ATP 5, Na-GTP 0.3, HEPES 10 (pH 7.20 adjusted by KOH). APs were recorded using the patch-clamp whole-cell configuration in current-clamp mode (*I*=0). Evoked APs were elicited by brief (1 ms) suprathreshold current pulse. Analysis of APs recordings was performed in software package ClampFit 10 (Molecular Devices, Sunnyvale, CA, USA). To characterize the L-type Ca^2+^-current we used the protocol described previously ^11^. The internal solution contained (in mmol/L): 110 CsOH, 90 aspartic acid, 20 CsCl, 10 tetraethyl ammonium chloride (TEA chloride), 10 HEPES, 10 EGTA, 5 Mg-ATP_2_, 5 Na_2_-creatine phosphate, 0.4 GTP-Tris, 0.1 leupeptin (pH 7.2 with CsOH) and the bath solution contained (in mmol/L): 125 *N*-methyl-glucamine, 5 4-aminopyridine (4-AP), 20 TEA chloride, 2 CaCl_2_, 2 MgCl_2_, 10 glucose and 10 HEPES (pH 7.4 with HCl). After an initial 1-sec prepulse at -40 mV, Ca^2+^-currents were elicited using 200-ms voltage steps from -30 to +50 mV in 10-mV increments. Voltage-dependence of inactivation was assessed by holding cells at various potentials from -40 to +10 mV for 2 sec followed by a 100-ms test pulse to +10 mV. The protocol for potassium currents measurement was described previously ^12^. The internal solution contained (in mmol/L): 110 K-aspartate, 20 KCl, 8 NaCl, 1 MgC1_2_, 1 CaC1_2_, 10 BAPTA, 4 K_2_ATP and 10 HEPES (pH 7.2 with KOH). The bath solution was Tyrode (see above). The K-current was elicited by a series of 500-ms voltage steps varying from -110 to +50 mV in 10-mV increments at 0.2 Hz frequency. The current density of the inward rectifier K-current (I_K1_) was determined at the end of the voltage steps, ranging from -110 to -40 mV. To eliminate the transient outward K-current (I_Kto_) an inactivating voltage prepulse was applied (50 ms, -40 mV). The remaining current consisted of the ultra-rapid delayed rectifier K-current (I_Kur_) and the steady-state outward K-current (I_Kss_). 100 µmol/L 4-AP (which blocks I_Kur_) was applied to record I_Kss_. The density of I_to_ was obtained by off-line subtracting the current traces measured with and without the inactivating prepulse, while I_Kur_ was measured as a subtraction of currents recorded in the absence and presence of 4-AP. The current densities of each of the three components of the outward K-currents were determined at the peak current. Holding current in all current clamp data was subtracted, we did not find significant differences in holding currents between groups. Mean values of holding currents shown in Suppl. Table S4.

## **Histology**

Immediately after euthanasia and blood sample collection from right ventricle of heart, the animal was perfused transcardially with 6 – 8 ml phosphate buffered saline (PBS pH 7.4, Gibco). The apex (one third of the heart) was separated and the rest of the heart was dissected and fixed in 4 % PFA solution (4 % paraformaldehyde, 2 mmol/L EDTA and 0.05 mmol/L BHT pH 7.4 adjusted with 1 mol/L HCl) for 20 hours in 4°C. After fixation, the tissue samples were stored in phosphate buffered saline (PBS pH 7.4, Gibco) at 4°C until further processing. After paraffin embedding, three cross-sectional 5 µm thick sections were cut with 200 – 300 microns distance from middle third of the heart. Masson´s trichrome staining (MTC) was used for assessing heart morphology of 12- and 16-week-old mice and representative section of each groups were imaged with microscope (Plan 1X UW objective, Nikon Eclipse Ni-e microscope, Nikon Instruments Europe B.V, manufactured in Japan). A standard hematoxylin and eosin based staining was used to stain the samples from 21-week-old mice and the section representing the midpoint of the heart was imaged with microscope (Nikon Plan Fluor 4X objective, Nikon Eclipse Ni-e microscope, Nikon Instruments Europe B.V, manufactured in Japan). The left ventricle wall thickness (mean of four different points: anterior, posterior, septum and lateral) and left ventricle diameter (inner perimeter derived circular diameter, papillary muscles were ignored) of the representing section were measured with NIS-Elements software (Nikon Instruments Europe B.V). The relative wall thickness (RWT) was calculated as RWT = (LV outer diameter - LV inner diameter)/LV inner diameter. For statistical analysis, 10 samples in each group were used.

## **Western Blot**

Total protein or nuclear fractions were isolated from left ventricular tissue samples as before ^6^. For detection of PGC-1α, 25 µg of nuclear protein was run on gradient SDS-PAGE gel and transferred to 0.2 µm nitrocellulose membrane. Primary antibody dilution of mouse anti-PGC-1α (4C1.3, Calbiochem, Darmstadt, Germany) and rabbit anti-Lamin B (ab16048, Abcam, Cambridge, UK) were incubated with the membrane overnight, 4 °C. For detection of 5' AMP-activated protein kinase (AMPK), 20 µg of total protein was run on 8 % SDS-PAGE gel and transferred to 0.2 µm nitrocellulose membrane. Total AMPK was detected with rabbit anti-AMPKα (SAB4502329, Sigma-Aldrich) and phosphorylated AMPK with rabbit anti-p-AMPKα1/2(Thr172) (sc33524, Santa Cruz Biotechnology) antibodies. Fluorescent Cy-labelled or HRP-conjugated secondary antibodies were incubated 1 h at room temperature and protein bands detected with GelDoc^TM^ MP imaging system (Bio-Rad Laboratories Inc., Hercules, CA, USA). Quantification of proteins was done with ImageJ software and Ponceau S staining (Sigma-Aldrich) was used as a loading control. Unedited blots are shown in Supplementary Figure S6.

## **Transverse tubule staining and analysis**

For t-tubular staining and morphological analysis, isolated cardiomyocytes were plated on laminin (L2020 Sigma-Aldrich) coated 35 mm glass bottom dishes (P35G-1.5-7-C MatTek Corp.) and incubated 45-75 min at 37°C and 5 % CO_2_. Before fixation, the cells were incubated 10 min at 37°C and 5 % CO_2_ with CellMask Orange Plasma Membrane (1:1000, C10045 ThermoFisher Scientific). The cells were rinsed two times with PBS and fixed with 4 % paraformaldehyde in PBS 10 min at room temperature. After fixation, samples were washed with PBS three times for two minutes at room temperature and stored in PBS. The stained samples were imaged immediately using a confocal microscope (Olympus Fluoview 1000, Japan). Oil immersion 100X objective, resolution 1600x1600 pix, excitation 568 nm and emission band 580 - 680 nm were used. Data were analyzed using MatchedMyo software ^13^. Briefly, MatchedMyo utilizes a matched-filter-based algorithm to detect regular t-tubules (TT-content), longitudinal tubules (LT-content) and T-system absence (TA-content). Additionally, cytosol area which did not fall into those categories was considered as irregular T-system (IT-content). To measure the relative area with irregular t-tubule we used simple calculation: IT-content = 100% - TT-content - TA-content - LT-content. Prior to the t-tubule analysis ImageJ software was used to exclude sarcolemma, nuclei and extracellular space from the analysis.

## **Statistical testing**

For statistical analysis OriginPro program was used (OriginLab Corporation, Northampton, MA, USA). Results are presented as mean ± SEM (standard error of the mean). Hierarchical linear model was used to correct potential clustering of data for all experiments utilized isolated cardiomyocytes ^14^. In other cases, statistical significance and interaction between sex and genotype was estimated with two-way ANOVA and statistical differences between individual groups were determined by Bonferroni post hoc test.

# **References**

1. Karkkainen O, Tuomainen T, Mutikainen M, Lehtonen M, Ruas JL, Hanhineva K, Tavi P. Heart specific PGC-1alpha deletion identifies metabolome of cardiac restricted metabolic heart failure. *Cardiovasc Res* 2019;**115**:107-118.

2. Kim D, Langmead B, Salzberg SL. HISAT: a fast spliced aligner with low memory requirements. *Nat Methods* 2015;**12**:357-360.

3. Hartley SW, Mullikin JC. QoRTs: a comprehensive toolset for quality control and data processing of RNA-Seq experiments. *BMC Bioinformatics* 2015;**16**:224.

4. Love MI, Huber W, Anders S. Moderated estimation of fold change and dispersion for RNA-seq data with DESeq2. *Genome Biol* 2014;**15**:550.

5. Huang da W, Sherman BT, Lempicki RA. Systematic and integrative analysis of large gene lists using DAVID bioinformatics resources. *Nat Protoc* 2009;**4**:44-57.

6. Mutikainen M, Tuomainen T, Naumenko N, Huusko J, Smirin B, Laidinen S, Kokki K, Hynynen H, Yla-Herttuala S, Heinaniemi M, Ruas JL, Tavi P. Peroxisome proliferator-activated receptor-gamma coactivator 1 alpha1 induces a cardiac excitation-contraction coupling phenotype without metabolic remodelling. *J Physiol* 2016;**594**:7049-7071.

7. Shannon TR, Ginsburg KS, Bers DM. Quantitative assessment of the SR Ca 2+ leak-load relationship. *Circulation Research* 2002;**91**:594-600.

8. Louch WE, Mork HK, Sexton J, Stromme TA, Laake P, Sjaastad I, Sejersted OM. T-tubule disorganization and reduced synchrony of Ca2+ release in murine cardiomyocytes following myocardial infarction. *J Physiol* 2006;**574**:519-533.

9. Heinzel FR, Bito V, Volders PG, Antoons G, Mubagwa K, Sipido KR. Spatial and temporal inhomogeneities during Ca2+ release from the sarcoplasmic reticulum in pig ventricular myocytes. *Circ Res* 2002;**91**:1023-1030.

10. Louch WE, Bito V, Heinzel FR, Macianskiene R, Vanhaecke J, Flameng W, Mubagwa K, Sipido KR. Reduced synchrony of Ca2+ release with loss of T-tubules-a comparison to Ca2+ release in human failing cardiomyocytes. *Cardiovasc Res* 2004;**62**:63-73.

11. Xu L, Li XY, Liu Y, Li HT, Chen J, Li XY, Jiang XJ, Wu G, Tang YH, Wang X, Huang CX. The mechanisms underlying ICa heterogeneity across murine left ventricle *Mol Cell Biochem* 2011;**352**:239-246.

12. Rivard K, Trepanier-Boulay V, Rindt H, Fiset C. Electrical remodeling in a transgenic mouse model of alpha1B-adrenergic receptor overexpression *Am J Physiol Heart Circ Physiol* 2009;**296**:H704-718.

13. Colli DF, Blood SR, Sankarankutty AC, Sachse FB, Frisk M, Louch WE, Kekenes-Huskey PM. A Matched-Filter-Based Algorithm for Subcellular Classification of T-System in Cardiac Tissues. *Biophys J* 2019;**116**:1386-1393.

14. Sikkel MB, Francis DP, Howard J, Gordon F, Rowlands C, Peters NS, Lyon AR, Harding SE, MacLeod KT. Hierarchical statistical techniques are necessary to draw reliable conclusions from analysis of isolated cardiomyocyte studies. *Cardiovasc Res* 2017;**113**:1743-1752.
